# Wheat Genotype-Specific Recruitment of Rhizosphere Bacterial Microbiota Under Controlled Environments

**DOI:** 10.3389/fpls.2021.718264

**Published:** 2021-12-01

**Authors:** Christine Jade Dilla-Ermita, Ricky W. Lewis, Tarah S. Sullivan, Scot H. Hulbert

**Affiliations:** ^1^Department of Plant Pathology, Washington State University, Pullman, WA, United States; ^2^Department of Crop and Soil Sciences, Washington State University, Pullman, WA, United States

**Keywords:** wheat, genotype, rhizosphere, recruitment, microbiome, Rhizoctonia

## Abstract

Plants recruit beneficial microbial communities in the rhizosphere that are involved in a myriad of ecological services, such as improved soil quality, nutrient uptake, abiotic stress tolerance, and soil-borne disease suppression. Disease suppression caused by rhizosphere microbiomes has been important in managing soil-borne diseases in wheat. The low heritability of resistance in wheat to soil-borne diseases like Rhizoctonia root rot has made management of these diseases challenging, particularly in direct-seeded systems. Identification of wheat genotypes that recruit rhizosphere microbiomes that promote improved plant fitness and suppression of the pathogen could be an alternative approach to disease management through genetic improvement. Several growth chamber cycling experiments were conducted using six winter wheat genotypes (PI561725, PI561727, Eltan, Lewjain, Hill81, Madsen) to determine wheat genotypes that recruit suppressive microbiomes. At the end of the third cycle, suppression assays were done by inoculating *R. solani* into soils previously cultivated with specific wheat genotypes to test suppression of the pathogen by the microbiome. Microbiome composition was characterized by sequencing of 16S rDNA (V1-V3 region). Among the growth cycling lengths, 160-day growth cycles exhibited the most distinct rhizosphere microbiomes among the wheat genotypes. Suppression assays showed that rhizosphere microbiomes of different wheat genotypes resulted in significant differences in shoot length (value of *p*=0.018) and had an impact on the pathogenicity of *R. solani*, as observed in the reduced root disease scores (value of *p*=0.051). Furthermore, soils previously cultivated with the *ALMT1* isogenic lines PI561725 and PI561727 exhibited better seedling vigor and reduced root disease. Microbiome analysis showed that Burkholderiales taxa, specifically *Janthinobacterium*, are differentially abundant in PI561727 and PI561725 cultivated soils and are associated with reduced root disease and better growth. This study demonstrates that specific wheat genotypes recruit different microbiomes in growth chamber conditions but the microbial community alterations were quite different from those previously observed in field plots, even though the same soils were used. Genotype selection or development appears to be a viable approach to controlling soil-borne diseases in a sustainable manner, and controlled environment assays can be used to see genetic differences but further work is needed to explain differences seen between growth chamber and field conditions.

## Introduction

The rhizosphere is a dynamic region of soil immediately surrounding plant roots that emerges through the interaction between plant roots, soil, and microorganisms (Hinsinger et al., 2009; Philippot et al., 2013). Rhizosphere-associated microbes have been documented to be involved in plant health (Berendsen et al., 2012; Pollak and Cordero, 2020).

Root-derived carbon makes the rhizosphere a hot spot for numerous microbial activities and interactions, affecting nutrient cycling, plant growth, and tolerance to abiotic and biotic stress (Philippot et al., 2013). Some work has shown that wheat plants can allocate up to 25% of total photosynthate to the roots; roughly, 13% is retained in the roots, 9% is respired by roots, and about 3% is retained in soil organic matter (SOM) and microbial biomass (Kuzyakov and Domanski, 2000). Root exudates are widely thought to be major sources of carbon release in the rhizosphere, but several other root processes can influence the rhizosphere carbon pool. For instance, roots can release mucilage and lysates, cells may slough off, and carbon can be released in the mycorrhizosphere by plant-associated mycorrhizal fungi (Dennis et al., 2010). Furthermore, root traits and architecture have been associated with differences in microbiome composition (Pérez-Jaramillo et al., 2017; Saleem et al., 2018).

While the relative contribution of these various aspects of root biology to rhizosphere community structure and functioning is an ongoing debate, evidence is growing that plant developmental stage and plant genotype can influence bacterial recruitment in the rhizosphere. For instance, microbiome structure has been shown to undergo successional changes with plant development, with the phenomenon being consistent across field trials (Walters et al., 2018). Additionally, co-occurrence network analyses have shown that rhizosphere communities become less diverse, but more tightly connected through the course of plant development (Shi et al., 2015).

Plant genotype has been shown to play a significant role in microbiome recruitment by various plants, including maize (Peiffer et al., 2013), barley (Bulgarelli et al., 2015), cotton (Qiao et al., 2017), common bean (Pérez-Jaramillo et al., 2017), and wheat (Mahoney et al., 2017). In fact, a large study with maize suggests microbiome structure could potentially be considered a heritable trait (Peiffer et al., 2013). In the inland Pacific Northwest, soil bacterial community structure and function have been clearly influenced by the genotype of field-grown winter wheat cultivars (Mahoney et al., 2017).

Genotype-specific recruitment of the microbiome is gaining interest with the idea that host genotypes will attract bacteria with specific outcomes in different agroecosystems. A study by Mazzola and Gu (2002) demonstrated that genotype-specific recruitment of specific fluorescent pseudomonads in wheat was associated with disease suppression against *Rhizoctonia solani* AG-5 and AG-8 in apple orchard soils. The induction of this kind of disease suppression by wheat genotypes could expedite the process as natural suppression of soil-borne pathogens typically takes years to develop in the field (Weller et al., 2002; Schillinger and Paulitz, 2014). Losses from rhizoctonia root rot (Rhizoctonia solani AG-8) in the Pacific Northwest are most clearly observed under minimum tillage or no-till system (Weller et al., 1986; Pumphrey et al., 1987), and bare patches are more prevalent in low rainfall areas than in high rainfall areas (Okubara et al., 2014). Manipulation of the soil microbiome could provide a novel sustainable approach to disease control.

Plant-driven manipulation of the microbiome requires the identification of desired host genotypes, which is a time intensive process that is compounded by the long length of time needed to observe resistance in the field. Optimization of growth chamber cycling experiments that generate genotype-specific microbiomes in wheat would facilitate the ease of doing microbiome structural/functional analyses, thereby shortening turn-around time in studying genotype-specific disease-suppressive wheat microbiomes. Thus, the first objective of our work was to determine whether the influence of plant genotype on rhizosphere microbiome recruitment observed by Mahoney et al. (2017) in field trials could be replicated under growth chamber conditions. Another objective of this study was to examine the influence of cycling length on genotype-specific microbiome recruitment. Ultimately, our work aims to identify wheat genotypes, along with their rhizosphere microbiomes, that are associated with improved plant health and reduced root rot disease caused by *R. solani* AG-8. Identification of wheat genotypes that recruit disease-suppressive microbiomes would further efforts to manipulate the rhizosphere for sustainably managing soil-borne diseases in wheat. Additionally, results from this study will identify useful parental genotypes for genetic studies on microbiome recruitment by wheat.

## Materials and Methods

### Soil Collection

Soils used for the growth chamber cycling experiments were collected from the Washington State University Plant Pathology Farm, Pullman, WA (46°46′38.0″N 117°04′57.4″W) in 2016 and 2017. These are the plots used by Mahoney et al. (2017). Soils at the site are classified as Palouse-Thatuna silt loam, characterized by moderately to well-drained soils (Donaldson, 1980), receiving an annual precipitation of roughly 53 centimeters. These soils have an average pH of 5.1 and aluminum (Al) concentration of 14.87ppm, based on recent soil tests (Soiltest Farm Consultants, Inc., Moses Lake, WA). The plot had been fallowed after a wheat crop in 2014. Soil collection was done in separate batches for the four growth chamber cycling experiments, specifically P28 (September 16, 2016), P35a (May 30, 2017), P35b (November 30, 2017), and P160 (September 1, 2017). The upper 25cm of soil across a transect from an experimental field were collected, sieved to 2mm, and homogenized. Afterward, soils were dispensed into 9cm^2^ pots (~400g soil) for 28-and 35-daycycles. For 160-daycycles, 13cm^2^ pots were filled with 2,000g of soil.

### Wheat Genotypes

The six winter wheat genotypes used in this study were a subset of the nine wheat varieties that previously exhibited distinct microbiomes in the field (Mahoney et al., 2017). Among the six genotypes were two near-isogenic lines carrying alleles of the *ALMT1* (Aluminum-activated Malate Transporter 1), namely, PI561725 (*ALMT1*-1) and PI561727 (*ALMT1*-2) in the Century background (Carver et al., 1993; Houde and Diallo, 2008). The other genotypes were the PNW soft white winter varieties, Eltan, Madsen, Hill81, and Lewjain (Mahoney et al., 2017).

### Growth Chamber Cycling

Experiments were conducted in the growth chambers of the Plant Growth Facility of Washington State University (Pullman, WA, United States). Seeds were surface-sterilized with 10% bleach for 5min and subsequently washed three times with sterile water before being pre-germinated at 10°C overnight and incubated at room temperature for another 24h. Pre-germinated seeds of the six winter wheat genotypes were sown into pots (5 seedlings per pot). Growth chamber conditions were 18°C at night and 22°C during the day, with 12-h light periods. Pots were watered every other day with 35ml tap water. To simulate seasonal planting, the same wheat genotype was grown in the same pot (same soil) three consecutive times (three cycles). Cycle length refers to the number of days the wheat seedlings or plants were grown in each cycle before they were removed and replanted with minimal soil disturbance. Soils were collected for microbiome studies after the third cycle of planting. Three cycling lengths, 28-, 35-, and 160-day were examined. Four growth chamber cycling experiments were performed in this study: 28-daycycles (P28); 35-daycycles Trial 1 (P35a); 35-daycycles Trial 2 (P35b); and 160-daycycles (P160). A randomized complete block design with eight replicates per wheat variety was implemented except for 28-daycycles with four replicates per wheat variety. At the end of each cycle, shoots of the plants were excised, and new pre-germinated seeds were sown for another cycle after a rest period of two days. For the 160-daycycles, a week after sowing, pots were transferred to a 4°C vernalization chamber for 56days to allow winter wheat to flower. Pots were watered with 70–100ml tap water in alternate days. Fertilization was done by diluting 20-10\u201320 fertilizer (Peters Professional, Summerville, SC) in water (150ppm) and watering plants with 70ml of the solution. Each cycle was terminated after genotypes reached reproductive stage and anthesis for the 160-daycycles.

### Rhizosphere Soil Collection and DNA Extraction

To compare the results of this current study to that of Mahoney et al. (2017), methods for rhizosphere soil collection and DNA extraction were performed as described previously. At the end of the third cycle, roots from three plants per pot were pooled after removing bulk soil and large soil aggregates. Pooled roots were placed in 50-mL centrifuge tube containing 20ml sterile water. Each tube was vortexed for 1min and then sonicated for 1min to collect the tightly bound rhizosphere soil. Using sterile forceps, roots were removed from each tube and then centrifuged at 10,000 *x g* for 5min. Supernatant was decanted carefully from the soil pellet, and 0.25g of soil pellet was used for DNA extraction using the PowerSoil DNA isolation kit (Mo Bio, Carlsbad, CA, United States). Rhizosphere genomic DNA extraction was performed following the protocol provided by the manufacturer, and DNA was stored at −80°C.

### Suppression Assay

*Rhizoctonia solani* AG-8 culture was cultured on potato dextrose agar (PDA) for one week. Pearl millet was autoclaved (121°C for 45min) twice, on consecutive days and was inoculated with PDA cubes of *R. solani*, grown for three weeks. The pearl millet inoculum was air-dried overnight on a kraft paper in a sterile laminar flow hood and was ground using a coffee grinder specifically used for *R. solani* AG-8. Ground pearl millet was then sieved using 2mm and 0.5mm sieves, and particles retained on the 0.5mm sieves were kept as inoculum. Inoculum was then enumerated on *Rhizoctonia* selective medium (Paulitz and Schroeder, 2005). Loosely bound soils from the third cycle of the 160-day growth chamber cycling experiment were used for the suppression assay. Half of the soil from each pot was inoculated with 100 propagules per gram (ppg) of the inoculum, while the other half was set aside for uninoculated control. Growth cones (Stuewe and Sons. Inc., Oregon, United States) were filled with 130g of soil (inoculated and uninoculated). Cones were then watered with 24ml of deionized water, covered with kraft paper, and allowed to reach equilibrium at 15°C for one week. Surfaced-sterilized seeds of the Alpowa spring wheat cultivar were pre-germinated (as described in the growth chamber cycling section) for two days, when the radicles from the seeds were 3–5mm long. Each cone was planted with two seeds and was covered with uninoculated soil (approximately 12mm layer). Plants were watered with 12ml of deionized water on alternate days. After 14days, when seedlings had at least two fully emerged leaves, shoot length and shoot weight were measured, and root disease severity was scored using a 0 to 8 scale described by Kim et al. (1997).

### Microbiome Sequencing and Data Analysis

Rhizosphere soil gDNA samples were sent to Molecular Research (MRDNA, Shallowater, TX, United States) for sequencing. The 16S rDNA gene (V1-V3 region) was amplified using barcoded forward (5′-AGRGTTTGATCMTGGCTCAG-3′) and reverse (5′- GTNTTACNGCGGCKGCTG −3′) primers (Lane, 1991; Kumar et al., 2011). Using the HotStart Taq Plus Master Mix Kit (Qiagen, United States), amplification was performed under the following conditions: 94°C for 3m, followed by 30cycles of 94°C for 30s, 53°C for 40s, and 72°C for 1m, with a final elongation step at 72°C for 5m. PCR products (amplicons) were checked for desired size and the relative intensity on 2% agarose gel. Samples were pooled together in equal proportions and were purified using calibrated Ampure XP beads (Illumina, San Diego, CA, United States). The DNA library was prepared using the pooled and purified PCR product and sequencing was done on Illumina MiSeq (Illumina, San Diego, CA, United States) following the manufacturer’s protocol. Raw sequence data were converted to. fastq files and de-multiplexed using the MR DNA software (MR DNA, Shallowater, TX, United States).

To directly compare operational taxonomic unit (OTU) IDs with that of Mahoney et al. (2017), raw sequence data from that study were processed along with the sequence data generated in the current study. Paired-end reads were processed in MICrobial Community Analysis (MICCA, version 1.6; Albanese et al., 2015). Merged and trimmed sequences were filtered by removing reads with an expected error rate of >0.5 and a length<400bp. Sequences were assigned to OTUs using an open-reference approach and the Greengenes reference database (ver.13.5) at 97% identity, and chimeric sequences were removed. Consensus classifier was used to classify OTU sequences using the Greengenes taxonomic references and was then aligned using nearest alignment space termination (NAST). These output files were then used to generate a. biom file (McDonald et al., 2012) for downstream analysis.

Analysis and visualization of microbiome data were performed in R statistical software (R Core Team, 2017) using the Phyloseq (McMurdie and Holmes, 2013) and ggplot2 (Wickham, 2009) packages. Non-bacterial OTUs and sequences that were classified as chloroplast or mitochondrial were removed from further analysis. The plot_richness function (Phyloseq) was used to assess alpha diversity. Furthermore, relative abundance of rarefied data was used to determine Bray-Curtis distances and ANOVA was performed to determine differences, and ordination was performed using CAP (Canonical Analysis of Principal coordinates; Anderson and Willis, 2003) in Phyloseq to determine genotype effects on the beta diversity of microbiome.

Significant differences in the microbiome of different wheat genotypes were assessed using relative abundance of unrarefied data was log(x+1) transformed and multivariate analysis of variance with permutation (PERMANOVA; Kelly et al., 2015) using a Bray–Curtis dissimilarity matrix (999 permutations) with PRIMER (v7, PRIMER-E, Plymouth, UK). Pairwise tests in PRIMER were performed after significant differences were determined among microbiomes of the six winter wheat genotypes. To further investigate genotype-specific effects on the microbiome, identification of differentially abundant (DA) OTUs was done using data from growth chamber cycling experiments that exhibited genotypic differences. Differentially abundant OTUs were identified through the Wald test of the DESeq2 package in R (Love et al., 2014). Unrarefied OTU data were filtered to remove low abundance taxa (<10 total counts) and those that have less than five counts in three samples after normalization based on geometric means. Differences in the abundance of OTUs were evaluated at *α*=0.1 using Benjamini-Hochberg adjusted values of *p*. Abundance of differential OTUs was then plotted in a heatmap using DESeq2 normalized log(x+1) transformed counts.

To determine whether the microbiome found in the growth chamber is comparable to the microbiome of the six wheat varieties in the field (Mahoney et al., 2017), dissimilarity matrices of growth chamber data sets and field data set (Mahoney et al., 2017) were generated using Bray-Curtis distance in the vegan package in R. A Mantel test (Legendre and Legendre, 2012) was then performed using Spearman correlation coefficients with 999 permutations.

Microbiome network analysis was performed using sparse inverse covariance estimation for ecological association inference (SPIEC-EASI; Kurtz et al., 2015). The top 205 taxa were selected using relative abundance and rarefied OTU tables, while the ecological network was calculated using unrarefied OTU tables (as required by SPIEC-EASI). SPIEC-EASI parameters were as follows: method=“mb” (Meinshausen and Bühlmann, 2006), lambda.min.ratio=1e-2, nlambda=100, and rep.num=100. Graphical interpretations of networks were visualized using the Fruchterman-Reingold layout. To highlight the strongest correlations between taxa, edges with an absolute weight<0.1 were removed. To directly determine network modularity and roles of differentially abundant OTUs in P160, relative abundance of all differentially abundant OTUs identified from DESeq2 were used for network analysis as well. Modularity within networks was examined *via* the rnetcarto package in R (Doulcier and Stouffer, 2015). Roles in the network structure were assigned to nodes belonging to specific modules (Guimerà and Amaral, 2005) with slight modification (Olesen et al., 2007) after generating consensus results from 20 iterations. The ggnet2 package was used to visualize the networks.^1^

Data obtained from the suppression assay were then tested for normality using a Shapiro–Wilk test (Shapiro and Wilk, 1965), and homogeneity of variance was examined using Levene’s test (Levene, 1960) through the car 2.1–6 package (Fox and Weisberg, 2011) in R. Since the data did not satisfy the assumptions of ANOVA, statistical significance among treatment means was determined using the non-parametric Kruskal-Wallis test (Kruskal and Wallis, 1952) with the agricolae 1.2–8 package (de Mendiburu, 2009) in R. Multiple comparison of treatment means was then done using kruskal function (Conover, 1999) using Fisher’s least significant difference criterium with *α*=0.05. To identify bacterial OTUs correlated with plant growth (shoot length and shoot weight) and reduced root disease, a correlation and linear regression test of the top 50 differentially abundant OTUs (absolute abundance) with shoot length, shoot weight, and root disease score were done in R (Wilkinson and Rogers, 1973; Chambers, 1992).

## Results

### Rhizosphere Microbiome and Wheat Genotypes

The 16S rDNA (V1-V3) sequencing generated 19,358,470 total reads for all data sets. After quality filtering, chimera removal, and removal of sequencing reads assigned to non-bacterial operational taxonomic units (OTUs), the remaining 14,662 OTUs were identified at 97% similarity.

Alpha diversity of the rhizosphere microbiome of the different wheat genotypes varied across different growth chamber cycling lengths (Supplementary Figure 1; Supplementary Table 1). There were significant differences in the alpha diversity among different growth chamber cycling experiments (*p*=<0.0001). On the other hand, there were no significant differences in the alpha diversity among genotypes except for the 160-daycycles (*p*=0.032).

**Figure 1:**
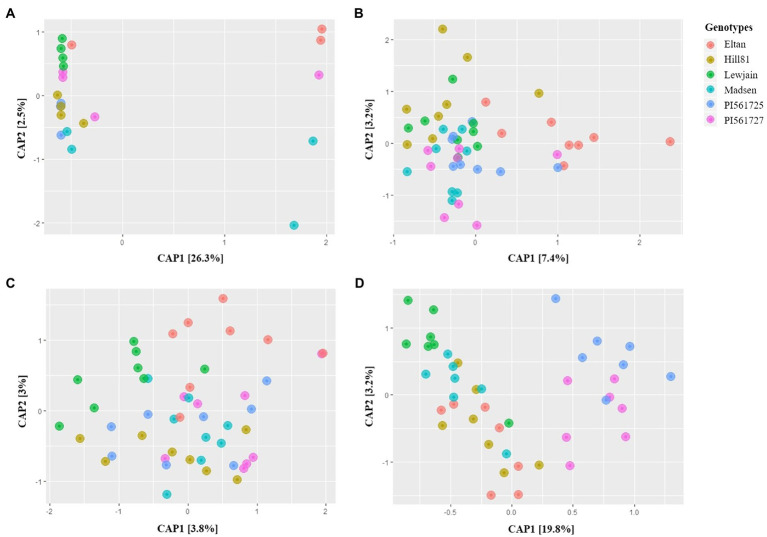
CAP plots of Bray-Curtis distances among bacterial communities of six winter wheat genotypes grown in Pullman soil under different growth chamber cycling lengths. **(A-D)** 28-, 35- (Trial 1), 35- (Trial 2), and 160-day cycle.

Rhizosphere bacterial microbiome of the six wheat genotypes grown in Pullman soil for the 28-daycycles (P28) did not show any significant differences (*p*=0.196) and no obvious clustering of the microbiome in the CAP ordination plots (Figure 1A). In contrast, the two different trials of 35-daycycles in Pullman soil, namely, P35a (*p*=0.002) and P35b (*p*=0.001), both exhibited significant differences in the microbiome among six winter wheat genotypes. Distinct separation of microbiomes of Eltan from Lewjain was observed in CAP plots for the two trials of 35-daycycles (Figures 1B,C). Pairwise PERMANOVA showed that there were statistical differences between Eltan and Lewjain in both P35a (*p*=0.038; Similarity=68%) and P35b (*p*=0.052; Similarity=65.46%; Supplementary Tables 2, 3). Furthermore, extending growth chamber cycles to reproductive stage at 160-daycycles (P160) resulted in clearly differentiated microbiome among wheat genotypes (Figure 1D; Supplementary Figure 2). In this trial, wheat genotypes accounted for 23% of variation in the composition of the microbiome based on the constrained ordination plot. There were significant differences in microbiome composition among the wheat genotypes (*p*=0.001) and obvious clustering of the ordination by genotype. Among 15 pairwise comparisons in PERMANOVA (Supplementary Table 2C), all pairwise comparisons done against the two *ALMT1* isogenic lines (PI561725 and PI561727) were significantly different (Supplementary Table 2C). However, these two isogenic lines were not significantly different from each other (value of *p*=0.4; Similarity=65.71%).

After filtering out OTUs with <0.001 relative abundance across all data sets, 4,593 bacterial OTUs belonging to 29 phyla were observed (Supplementary Figure 3). Among the bacterial OTUs, those belonging to Proteobacteria were predominant in all four growth chamber cycling trials (Supplementary Figure 3; Supplementary Table 4), followed by Bacteroidetes and Actinobacteria. Variation in abundance of specific bacterial phyla is evident among different wheat genotypes. For instance, at P28, Actinobacteria is more abundant in Eltan (26.1%) compared to the rest of the genotypes. However, as growth cycle length progressed, relative abundance of Actinobacteria decreased not only in Eltan, but among other wheat genotypes as well (Supplementary Table 4). As for Acidobacteria, abundance of this phyla increased as growth cycle length progressed. At P160, variation in the relative abundance of Acidobacteria among genotypes was apparent, with the two *ALMT1* isogenic lines (PI561725 and PI561727) having significantly lower abundance (10.1% average) while the rest of the genotypes averaged 14.4%. Conversely, Proteobacteria were consistently dominant across all four growth chamber cycling experiments and declined in relative abundance with increased growth chamber cycling length (from 47% in P28 to 33% in P160).

Differentially abundant (DA) OTUs among different wheat genotypes were identified in trials P35a, P35b and P160. However, differential OTUs for each wheat genotype were generally inconsistent between experiments and even between the two trials of 35-daycycles (P35a and P35b). Abundance of REF3578 (Oxalobacteraceae) was differentially increased in PI561725 compared with Eltan in both P35a and P160 (Supplementary Table 5; Supplementary Figure 4) but was not differential in P35b. In P35a, another bacterial OTU (REF2162) belonging to Oxalobacteraceae was differentially higher in PI561727 and lower in Lewjain, but in P35b this same OTU was differentially higher in Eltan compared to Hill81. The abundance of REF6703 (Sphingomonadaceae) had the opposite trend in P35a and P35b; it was differentially lower in PI561725 compared to Eltan in P35a but was higher in PI561725 than Eltan in P35b.

Several DA OTUs belonging to the same bacterial families exhibited differential enrichment in the rhizosphere of specific wheat genotypes. For instance, eight (DENOVO1204, DENOVO1885, DENOVO2591, DENOVO787, REF5077, REF591, REF6907, REF994) out of 14 DA OTUs belonging to Chitinophagaceae were differentially higher in Eltan than most of the winter wheat genotypes (Supplementary Table 5). Meanwhile, three DA OTUs belonging to Burkholderiaceae were consistently higher in PI561725 compared with Hill81 (REF2457) and Lewjain (DENOVO37, REF5019) in P35a. OTUs belonging to Sphingobacteriacea*e* (REF6072, REF7015, REF4083) were more enriched in PI561725 compared with Eltan (P35a) and Hill81 (P35b). Notably, four out of eight DA OTUs from Oxalobacteraceae (genus *Janthinobacterium*) were differentially higher in PI561725 than Eltan, Hill81, Lewjain, and Madsen in P35a, P35b and P160 (Supplementary Table 5; Supplementary Figure 4).

**Figure 2:**
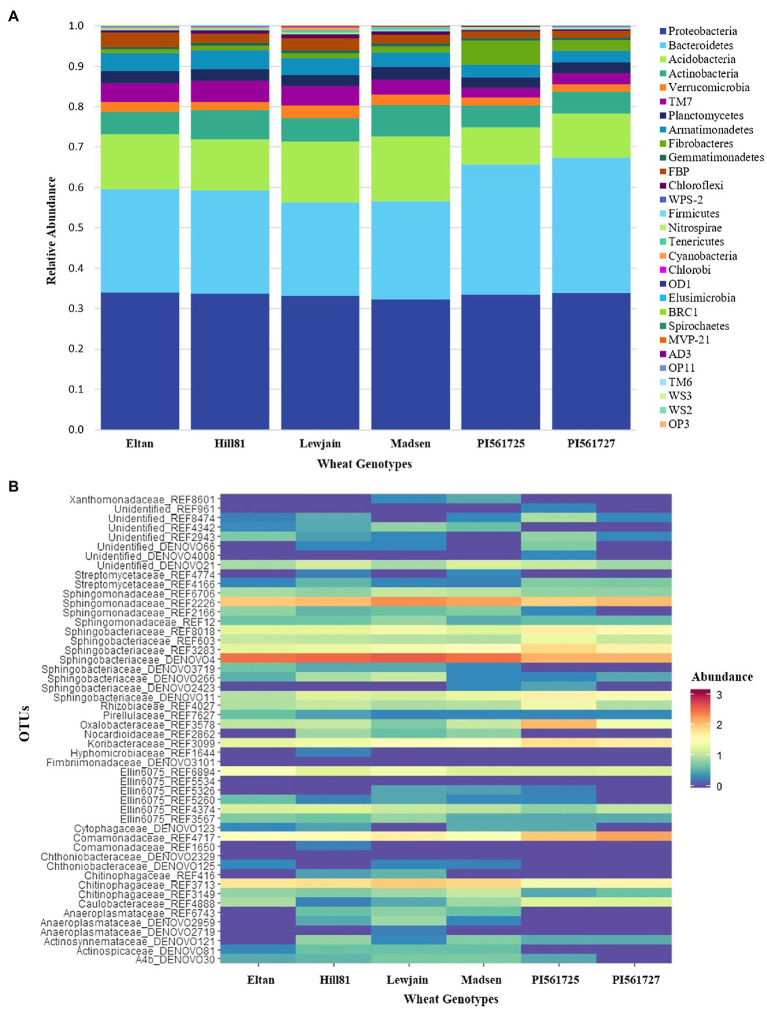
Abundant bacterial OTUs in six winter wheat genotypes grown on Pullman soil for three 160-daycycles. **(A)** Relative abundance of most abundant families. **(B)** Heatmap of normalized log (x+1) transformed counts of the top 50 differentially abundant OTUs across wheat genotypes generated from DESeq2.

The separation of the microbiome of the two *ALMT1* isogenic lines, PI561725 and PI561727, from the rest of the winter wheat genotypes in the extended cycle (P160) was attributed to 27 differentially abundant OTUs (Supplementary Table 5; Figure 2). In comparison with the rest of the wheat genotypes, five Sphingobacteriaceae OTUs were differentially higher in the *ALMT1* isogenic lines. Bacterial OTUs from Oxalobacteraceae (REF3578) and Comamonadaceae (REF4717) were more abundant in the two *ALMT1* lines compared with other four winter wheat genotypes. A streptomycete (REF4166) was differentially enriched in PI561725 and PI561727 compared to Eltan, Lewjain, and Madsen. On the other hand, Anaeroplasmataceae (DENOVO2959, REF6743), Actinospicaceae (DENOVO81), Chitinophagaceae (REF3713), and Sphingobacteriaceae (DENOVO266) were less abundant in the *ALMT1* lines compared with the other genotypes.

### Comparison of Growth Chamber and Field Rhizosphere Microbiome Composition

The microbiomes of each of the four growth chamber experiments were compared to that of the microbiomes described in the field experiments of Mahoney et al. (2017). The collective microbiome of the six winter wheat genotypes derived from the field study was significantly different from all growth chamber cycling experiments (value of *p*=0.001; Supplementary Table 2; Supplementary Figure 2). As wheat roots were harvested in P160 at the same growth stage as that of the field experiment, it was assumed that most likely the microbiome of the wheat genotypes in P160 and field would be more correlated. However, correlation analysis showed a low correlation with P160 (Spearman *r*=0.03; value of *p*=0.41). When microbiomes from the individual cycling experiments were individually compared to the field microbiomes, all four comparisons showed low similarities ranging from 33.45% for P35b to 31.79% for P28.

Microbiomes of the six winter wheat genotypes were also compared among different growth chamber cycling experiments. Pairwise PERMANOVA tests have shown that all growth chamber cycling experiments have significantly different microbiomes (value of *p*=0.001), even between the two trials of 35-daycycles (Supplementary Table 2, 3).

### Network and Network Roles in Different Wheat Genotypes

Analysis of the 205 OTUs with the highest relative abundance showed that Proteobacteria and Bacteroidetes generally dominated the ecological networks across all cycle lengths, and in the field (Supplementary Figure 5). Additionally, Acidobacteria were found to cluster throughout the networks, and Actinobacteria composed a larger cluster in the 35-day Trial 1 ecological network. Other phyla were present throughout the networks, including Armatimonadetes, Chloroflexi, Cyanobacteria, FBP, Fibrobacteres, Gemmatimonadetes, Planctomycetes, Tenericutes, TM7, and Verrucomicrobia. The highest number of edges were observed in the networks of the 35-daycycles (before and after edge filtering), with the 28-daycycle and the two-yearcycle resulting in the lowest number of edges before filtering (Supplementary Table 6). The lowest number of edges after filtering was observed in the 160- day cycle followed by the 28-daycycle. Both before and after edge filtering, ecological networks of all cycle lengths and the field were dominated by positive associations. After edge filtering, positive correlations were a higher percentage of the total edges compared to before edge filtering. In the growth chamber studies, the 28-daycycle had the lowest number of positive associations after edge filtering, and all networks from the growth chamber had a higher percentage of positive associations than the field study (before and after edge filtering).

Because a clear genotype-driven differentiation was observed between microbiomes in the 160-daycycle, the ecological networks were compared across genotypes using only the differentially abundant taxa (the top 205; Supplementary Figure 6). The same phyla that dominated the ecological networks of the rhizosphere microbiome when compared by cycle length also dominated the ecological networks of the rhizosphere microbiome when assessed by genotype. However, there were genotype-dependent differences in the number of phyla present in the ecological network and in the percentage of positive associations. Madsen had the most diverse networks in terms of number of phyla in the network, while Hill81 and the two isogenic lines had less diverse ecological networks. In terms of positive associations, the Madsen and PI561725 rhizospheres had the highest, and the Lewjain rhizosphere had the lowest, compared with all other genotypes (prior to edge filtering; Supplementary Table 7). After edge filtering, a similar trend was observed in terms of the percentage of positive associations, with PI561725 rhizospheres having the highest, and the PI561727 rhizosphere having the lowest, compared with all other genotypes.

To better understand the interactions within the microbiome assemblage in each genotype network, differentially abundant OTUs in P160 were partitioned to different modules and were assigned to different network roles Guimerà and Amaral (2005); Olesen et al. (2007). Assemblage of OTUs in network modules varied among different wheat genotypes (Supplementary Table 7) and assumed unique roles within and among modules (Supplementary Figure 7). The individual genotype network structure was partitioned to six roles, namely, module hub, peripheral hub, connectors, peripheral, ultra-peripheral, and kinless nodes (Supplementary Table 7). For module hubs, characterized by nodes linking OTUs within each module, only few were found. Module hubs in Lewjain, namely DENOVO37 (Proteobacteria) and REF4973 (Acidobacteria) were found (Supplementary Figure 7c). While REF115 (Fibrobacteres) was the only module hub in PI561725 (Supplementary Figure 7E; Supplementary Table 7). For the rest of the genotypes, there were no OTUs that had enough links within a module to be considered as a module hub (>2.5 within module degree; Poudel et al., 2016). On the other hand, connector nodes, those that have more links to OTUs from other module (Guimerà and Amaral, 2005), were detected among different individual genotype networks. These connector nodes are important to network coherence as they connect modules together (Olesen et al., 2007). Among the six wheat genotypes, PI561725 had the most connector nodes, while PI561727 and Lewjain had the least (Supplementary Figure 7; Supplementary Table 7). Most of the OTUs in the networks were either classified as peripheral nodes which are characterized by most links within same module or ultra-peripheral nodes which are only linked to nodes within the same module (Guimerà and Amaral, 2005). Both PI561725 and PI561727 had the least number of ultra-peripheral nodes, while Madsen had the greatest number of ultra-peripheral nodes (Supplementary Table 7). Furthermore, some of the differentially abundant OTUs included in the network had exhibited different roles among the individual network of the six wheat genotypes (Table 1).

**Table 1:** Summary of the suppression assay after the third cycle of the 160-day growth cycle.

Genotype	Uninoculated				100 ppg of *R. solani* AG8					
	Shoot Length		Shoot Weight		Shoot Length		Shoot Weight		Root Score^*^	
	Mean	Standard Deviation	Mean	Standard Deviation	Mean	Standard Deviation	Mean	Standard Deviation	Mean	Standard Deviation
Eltan	16.60^c^	0.87	0.201^bc^	0.03	12.21^c^	2.12	0.111^bc^	0.03	6.36^a^	0.94
Hill81	15.15^c^	2.84	0.186^c^	0.04	13.93^abc^	2.45	0.131^abc^	0.03	6.33^ab^	0.75
Lewjain	16.29^c^	1.89	0.184^c^	0.04	14.89^ab^	1.08	0.141^ab^	0.03	5.50^bc^	0.71
Madsen	16.76^bc^	1.25	0.205^bc^	0.03	12.83^bc^	3.74	0.107^c^	0.04	6.43^ab^	1.10
PI561725	18.49^a^	1.02	0.267^a^	0.03	15.81^a^	1.94	0.149^a^	0.03	5.36^c^	0.90
PI561727	17.88^ab^	2.26	0.238^ab^	0.04	15.91^a^	1.31	0.136^abc^	0.02	5.43^c^	0.45

* *Root score using 0–8 scale (0-no lesion; 8-severe lesion with almost no root growth). Different letter annotations in the means of each genotype indicate statistical significance (using Fisher’s least significant difference α=0.05).*

### Microbiome and Wheat Disease Suppression

To determine whether the wheat varieties showed differences in their abilities to recruit microbes that contribute to disease suppression, soils from one of the cycling experiments were used in a disease suppression assay. Soils from the experiment with longest (160-day) cycles were used because the microbiomes of different genotypes had differentiated better than in the shorter cycling experiments and suppressive soils generally take time to develop in field soils. Soils cultivated with the six winter wheat genotypes had variable effects on plant health when inoculated with *R. solani* and planted with the cultivar Alpowa (Table 1). Cultivation of Alpowa for 14days likely altered the microbiome to some extent but difference between the treatments should be due to the legacy effects of the genotypes used in the cycling experiment. There were significant differences in shoot length (value of *p*=0.018) and less impact on shoot weight (value of *p*=0.105) and root disease (value of *p*=0.051). Significant differences were also found in shoot length (value of *p*=0.008) and shoot weight (value of *p*=0.004) when Alpowa was planted in these soils without inoculum added. Based on *post hoc* tests, the soils previously cultivated with the *ALMT1* isogenic lines PI561725 and PI561727 outperformed most of the winter wheat genotypes except for Lewjain, in terms of shoot length and reduced root disease (Table 2). Soils cultivated with Madsen performed most poorly when inoculated with the pathogen.

**Table 2:** Differentially abundant OTUs correlated with traits in suppression assays and with relevant network roles.

OTUs	Genus	Correlated Traits^a^	Network Role × Module per Genotype^b^
REF3578	*Janthinobacterium*	Positively correlated with reduced root disease, shoot length, and shoot weight	Connector (PI561725), Ultra peripheral (PI561727, Madsen, Lewjain, Hill81, Eltan)
REF4717	Variovorax	Positively correlated with reduced root disease, shoot length, and shoot weight	Peripheral (PI561725, PI561727), Ultra Peripheral (Madsen, Lewjain, Hill81), Connector (Eltan)
REF1650	Unidentified	Positively correlated with root disease and negatively correlated with shoot length	
REF2166	Sphingomonas	Positively correlated with root disease	Peripheral (PI561727), Ultra peripheral (Madsen, Lewjain, Hill81, Eltan)
REF8601	Pseudoxanthomonas	Positively correlated with root disease	Ultra-peripheral (Lewjain, Hill81)
REF4166	Streptomyces	Positively correlated with	
shoot length and shoot weight	Peripheral (PI561725)		
REF603	Unidentified	Positively correlated with shoot length and shoot weight	Peripheral (PI561725, PI561727), Connector (Madsen), Ultra-peripheral (Lewjain, Hill81, Eltan)
REF8018	Unidentified	Positively correlated with shoot length and shoot weight	Connector (PI561725, Lewjain, Eltan), Peripheral (PI561727), Ultra-peripheral (Madsen, Hill81)
REF3283	Unidentified	Positively correlated with shoot length and shoot weight	Peripheral (PI561725, PI561727), Ultra-peripheral (Madsen, Lewjain, Eltan), Connector (Hill81)
REF3099	Candidatus Koribacter	Positively correlated with shoot length and shoot weight	Peripheral (PI561725), Connector (PI561727, Hill81, Eltan), Ultra peripheral (Madsen, Lewjain)
DENOVO2423	Unidentified	Positively correlated with shoot length and shoot weight	Ultra-peripheral (PI561725)
DENOVO11	Unidentified	Positively correlated with shoot length and shoot weight	Peripheral (PI561725, PI561727), Ultra peripheral (Madsen, Lewjain, Hill81, Eltan)
REF6743	Asteroleplasma	Negatively correlated with increased shoot weight (Uninoculated only)	Connector (Madsen, Eltan), Peripheral Hub (Lewjain), Ultra peripheral (Hill81)
REF4027	Unidentified	Positively correlated with shoot length and shoot weight (Uninoculated only)	Connector (PI561725, Madsen), Peripheral (PI561727), Ultra Peripheral (Lewjain, Hill81, Eltan)
REF961	Unidentified	Positively correlated with shoot weight (Uninoculated only)	
DENOVO2719	Asteroleplasma	Negatively correlated with increased shoot weight (Uninoculated only)	
DENOVO4	Unidentified	Negatively correlated with increased shoot weight (Uninoculated only)	Peripheral (PI561725), PI561727(Peripheral Hub), Ultra peripheral (Madsen, Lewjain, Hill81, Eltan)
DENOVO2959	Asteroleplasma	Negatively correlated with increased shoot weight (Uninoculated only)	Ultra-peripheral (Madsen, Lewjain, Eltan)
DENOVO81	Unidentified	Negatively correlated with increased shoot weight (Uninoculated only)	Ultra-peripheral (Lewjain, Hill81)
DENOVO828	Chryseobacterium	Positively correlated with shoot length and shoot weight (Uninoculated only)	Connector (PI561725), Ultra peripheral (PI561727, Eltan)
REF115	Unidentified	Positively correlated with shoot length, and shoot weight (Uninoculated only)	Module Hub (PI561725), Ultra-peripheral (PI561727, Lewjain, Madsen, Hill81, Eltan)
DENOVO37	Burkholderia	Positively correlated with reduced root disease; Increased shoot length, and shoot weight (Uninoculated only)	Connector (PI561725), Module Hub (Lewjain), Peripheral (PI561727), Ultra-peripheral (Madsen, Eltan)

a *Traits based on suppression assay identified to be correlated with OTU abundance.*

b *Network roles of OTUs* *(Guimerà and Amaral 2005)*; *Olesen et al. (2007)* *in corresponding module per genotype.*

Correlation analysis of shoot length, shoot weight, and root disease score with abundance of DA OTUs revealed specific bacterial orders associated with these traits in inoculated and uninoculated soils (Table 2). In soils inoculated with *R. solani* AG-8, REF3578 (Oxalobacteraceae) and REF4717 (Comamonadaceae), both of which belong to order Burkholderiales, were associated with both reduced root disease score, higher shoot length and shoot weight. In addition, these OTUs were also positively correlated with higher shoot length and shoot weight in uninoculated soils. In contrast, abundance of REF1650 (Comamonadaceae) was positively correlated with disease severity (Spearman *r*=0.31) and negatively correlated with shoot length (Pearson *r*=−0.48; Supplementary Table 8). Similarly, low abundance of REF2166 (Sphingomonadaceae) and REF8601 (Xanthomonadaceae) was associated with higher disease scores and lower shoot length. Higher shoot length was positively correlated with abundance of five OTUs from Sphingobacteriaceae, one Streptomycetaceae and one Koribacteraceae in inoculated soils (Supplementary Table 8). However, abundance of OTUs belonging to Rhizobiaceae (REF4027) and Weeksellaceae (DENOVO828) was only positively correlated with higher shoot length and shoot weight under uninoculated soils.

## Discussion

Microbes of the microbiome have drawn a great deal of attention in recent years, and studies have begun focusing on manipulating these microbiomes in order to strengthen sustainable agricultural systems. Factors, such as soil type (Qiao et al., 2017), plant growth stage (Chaparro et al., 2014; Yuan et al., 2015; Qiao et al., 2017; Walters et al., 2018), root system architecture (Saleem et al., 2018), and genotype (Micallef et al., 2009; Mahoney et al., 2017), have been documented to strongly influence the composition and function of the microbiome. In order to effectively manage the microbiome, we must first understand the plant factors that help control the assemblage and function of the microbiome. These factors include a complicated and dynamic role of host genotype and plant physiological stage of development. Our study has taken strides toward a greater understanding of the impacts of both factors in the recruitment of wheat rhizosphere microbiomes. Further, we were able to associate these factors with varying degrees of root disease suppression and severity, caused by the pathogen *R. solani* AG-8.

Rhizosphere microbiome recruitment of six winter wheat genotypes under growth chamber conditions were found to be genotype-specific, in agreement with what has been demonstrated in the field study conducted by Mahoney et al. (2017). In the current study, the most abundant phyla (Proteobacteria, Bacteroidetes, and Actinobacteria) among all growth chamber experiments were the same as the top three phyla in the wheat core rhizosphere microbiome identified by Mahoney et al. (2017). However, at lower taxonomic levels of OTUs, the microbiome composition of the wheat genotypes in the growth chamber cycling experiments was different when compared with the field study. This held true despite obtaining soils from the site where the field study was conducted. Additionally, different trends in genotype-specific microbiome selection were observed in the field compared with the growth chambers. For instance, the microbiome of PI561725 was distinctly different from PI561727 in the field (Mahoney et al., 2017), but this was not observed in any of the cycling experiments in the growth chamber. This distinctness of the microbiome found between the field and growth chamber cycling experiments might be explained by the seasonal variations in the soil microbiome (Li et al., 2020) during the time of soil collection or even the general difficulty in replicating field studies in controlled environments (and vice versa). In addition, these isogenic wheat lines had grown in the field for seven months, allowing more time to recruit distinct microbiomes compared with the same wheat isolines grown in the growth chamber cycles for only 160days in each cycle prior to rhizosphere soil collection. Thus, a variety of spatio-temporal, climatic, and plant physiological variables likely account for the observed differences in the microbiome described in the field versus those recruited from the same soils but described under controlled, greenhouse growth chamber conditions. This would seem to highlight the intrinsic difficulties in attempting to replicate field studies under controlled conditions. However, the data from the current study were compelling in many ways more closely related to the fundamental aspects of soil disease suppression.

In this study, we were able to directly relate rhizosphere microbiome recruitment specificity with the length of the cultivation cycles and/or physiological stage of the plant. With increasing growth chamber cycling lengths, greater differentiation of rhizosphere microbiomes across the six winter wheat genotypes was clear. In rhizosphere soils collected from the 28-daycycles (P28), the microbiome of the six wheat genotypes did not show any significant differences. However, genotype-specific recruitment of the rhizosphere microbiome became significant in the 35-daycycles for both trial 1 and trial 2. Genotypic effects are most notable in P160 when wheat genotype accounted for 23% of microbiome variation (Figure 1). In the 160-day cycling experiments, most of the wheat genotypes reached reproductive stage, and the *ALMT1* isogenic lines were already in the grain filling stage. Edwards et al. (2015) have demonstrated that genotypes significantly impact rhizosphere microbiome and showed that changes in the microbiome are correlated with developmental stages in rice. Similarly, rhizosphere microbiome was strongly influenced by plant age, followed by field, and then plant genotype in maize (Walters et al., 2018). Furthermore, the trend observed in our study agrees to what Schlemper et al. (2017) observed in the rhizosphere microbiome of sorghum where genotypic effects became more significant as plant transitions from vegetative to reproductive stage. This observation was similar to several reports that the reproductive stage of the plant has stronger selective influence on the rhizosphere microbiome compared to vegetative stage (Smalla et al., 2001; Inceoğlu et al., 2010; Walters et al., 2018).

Additionally, in the P160 cycling experiment, the two *ALMT1* (aluminum-activated malate transporter) isogenic lines (PI561725 and PI561727) clustered in the opposite plane of the other four winter wheat varieties (Figure 1). Although these two isogenic lines appear to be distinct from the rest of the wheat genotypes, there are no significant differences in the rhizosphere microbiomes of PI561725 and PI561727. These two lines differ in aluminum (Al) toxicity tolerance, PI561725 being tolerant, while PI561727 is susceptible (Carver et al., 1993). Houde and Diallo (2008) have characterized these lines to identify candidate genes underlying tolerance, and the *ALMT1* gene was a major gene associated with tolerance. With 50μm (~13ppm) of Al, malic acid excretion increased in Al tolerant lines (Delhaize et al., 1993) and gene expression of the *ALMT1* gene was observed in this concentration of Al (Sasaki et al., 2004). Accounting for the Al concentrations in the Pullman soil (14.87ppm DTPA extractable Al) based on soil test, concentrations in Pullman soil are slightly elevated. But accounting other edaphic factors in Pullman soils, taken together may have confounding effects, making this level of aluminum not enough to induce differentiation of the rhizosphere microbiome between isogenic lines. Despite the insignificant difference in taxonomic composition of these isogenic lines, looking closely at the assemblage of microbiome networks between these two isogenic lines, PI561725 selects for a greater percentage of positive associations among OTUs in the rhizosphere compared with PI561727. This suggests that taxa composition together with the assemblage of network structure may provide more meaning on how the genotype-specific microbiome function as a whole.

Despite significant differences in the rhizosphere microbiome composition in wheat genotypes between the two trials of 35-daycycles, soils previously cultivated with PI561725 in the suppression assays (data not shown) consistently bested the rest of the genotypes as observed in P160. This highlights the possibility that, despite the variation in the taxonomic composition of genotype-specific rhizosphere microbiome among experiments, there might be some community function that is being maintained regardless of the taxonomic shifts. Microbiome studies on bromeliads (Louca et al., 2016), bioreactors (Fernandez et al., 1999), and the human gut (The Human Microbiome Project Consortium, 2012) have shown that despite taxonomic variation, functional structure at the community level is relatively constant. Hence, it is important to account for the community function being maintained in studies that involved comparison of temporal variations in the microbiome.

Our study also confirmed differential recruitment of bacterial OTUs at the family level among wheat genotypes. Differences in the most abundant bacterial families in different growth chamber cycling lengths and wheat genotypes reflect the succession of microbial communities in each plant growth stage and differential recruitment of the microbiome of wheat genotypes. Identification of DA OTUs of wheat genotypes between different growth chamber cycling experiments demonstrated that each wheat genotype has a distinct set of DA OTUs, specific to plant physiological development and environmental conditions. However, looking at the family level of the DA OTUs in each wheat genotype, specific bacterial families are differentially recruited by specific wheat genotypes. For instance, eight DA OTUs belonging to Chitinophagaceae were differentially higher in Eltan, while the majority of the DA OTUs belonging to Burkholderiaceae and Oxalobacteraceae were differentially higher in PI561725 among different experiments.

The microbiome of each wheat genotype became more tightly regulated and conserved as plant maturity advanced. Alpha diversity and microbial network associations decreased as growth cycling length increased. Among the three growth chamber cycling lengths used in our study, P160 has the lowest alpha diversity indices. This observation was similar to what Shi et al. (2015) reported, where alpha diversity in the rhizosphere microbiome of *Avena fatua* decreased gradually through time as plant growth progressed. One explanation is that the more diverse non-rhizosphere soil population takes time to transform as bacteria acclimate to rhizosphere conditions. It is also apparent that rhizosphere conditions change as the plant matures. Chaparro et al. (2014) observed high sugar levels in the root exudates of Arabidopsis during the vegetative stage, which declines at the reproductive state, at which point, exudate concentrations of amino acids and phenolics increased. Thus, during early stages of growth, the plant attracts a wider range of metabolically diverse microorganisms in the soil compared to later growth stages (Chaparro et al., 2014). Furthermore, network analysis showed that the total number of edges and the percentage of positive associations was highest in the 35-daycycles. After edge filtering, the lowest total number of edges was observed in the 160-daycycles. Together these results suggest that the dynamics within the microbial community lead to an increased total number of associations early on in plant development, but that a lower number of associations reflects a narrowing niche with fewer available substrates at or near the seed-filling stage.

When challenged with the root rot pathogen *R. solani* AG-8, the above-described differential recruitment of specific bacterial OTUs by specific wheat genotypes translated to very specific plant responses in terms of plant growth and disease severity. Soils previously cultivated with the *ALMT1* isogenic lines (Carver et al., 1993) exhibited higher shoot length, shoot weight, and reduced root rot disease, while the opposite was observed in Madsen-cultivated soils. Correlation analysis of abundance of DA OTUs and traits measured in the suppression assays identified OTUs that were positively correlated with reduced root disease, increased shoot length and shoot weight, strongly suggesting organisms within these OTUs may play a role in disease suppression or resistance. Five OTUs belonging to Sphingobacteriaceae, one Streptomycetaceae, and one Koribacteraceae were positively correlated to higher shoot length and were differentially more abundant in the rhizosphere soils of both isogenic lines. Plant growth promotion, especially in inoculated soils, is an important microbial function for biological control agents. Sphingobacteriaceae (Morais et al., 2019) and Strepto-mycetaceae (Dias et al., 2017; Vurukonda et al., 2018) have been previously reported to exhibit plant growth promotion. Multiple mechanisms have been postulated for plant growth promotion, including phosphate solubilization (Rodríguez and Fraga, 1999; Compant et al., 2010), iron sequestration through siderophore production (Scagliola et al., 2016), and phytohormone modulation (de Garcia Salamone et al., 2005; Glick et al., 2007). Additionally, several bacterial OTUs were identified to play dual roles in plant disease suppression and plant growth promotion. Two OTUs belonging to order Burkholderiales were positively correlated with greater shoot length and shoot weight, and reduced root disease score. REF3578 (Oxalobacteraceae) and REF4717 (Comamonadaceae) were positively correlated with these traits and were differentially higher in PI561725 and PI561727 compared to the other four wheat genotypes. Recently, Yin et al. (2021) identified a species of *Janthinobacterium* from wheat rhizosphere soil associated with seedling tolerance to R. solani AG-8 after 5–6 growth cycles in the greenhouse. This genus was the same as the genus of REF3578 that was identified in our study associated with disease suppression and plant fitness. Bacterial species belonging to the Burkholderiales order have been reported to be associated with damping-off pathogen suppression in tomato and soybean (Benítez and Gardener, 2009). In addition to suppression, Burkholderiales species were considered plant growth-promoting, phosphate-solubilizing rhizobacteria (Goldstein, 1986; Rodríguez et al., 1996).

The identification of DA OTUs correlated with plant growth and reduced root disease leads to another question. What roles do the organisms within each of these OTUs play in the microbiome dynamics in each genotype? To further understand these interactions, network roles were determined (Guimerà and Amaral, 2005; Olesen et al., 2007). From the standpoint of ecological network structure, network hubs, connectors, and module hubs are network nodes that may have importance in maintaining a network (Poudel et al., 2016). Among the correlated DA OTUs in question, there were no network hubs, only module hubs and connectors. A module hub (REF115) in PI561725 network was detected and has been positively correlated with increased shoot length and shoot weight in uninoculated soils. Another important module hub in Lewjain was DENOVO37, which positively correlated with reduced root disease. The detection of these module hubs solidifies the roles of these correlated OTUs in the function of the microbiome network in terms of better plant growth and reduced root disease. These module hubs may facilitate the stable occurrence of other taxa and may serve as keystone taxa that support the co-occurrence of other organisms with desirable functional attributes (Poudel et al., 2016). Moreover, traits correlated OTUs identified as connector nodes were detected. Among the 40 connector nodes in PI561725, REF3578 (Oxalobacteraceae) was differentially higher in PI561725 and was correlated with better plant growth and reduced root disease. Most of the OTUs identified to be connectors were correlated with two or three traits in the suppression assays. Connector taxa are important to network structure as they provide links to other modules (Guimerà and Amaral, 2005) and may represent multi-functional taxa (Poudel et al., 2016). These results suggest that these OTUs are relevant in maintaining a specific function in the wheat microbiome and may be good candidates for more downstream functional analyses.

The current study demonstrated that the wheat microbiome involved in plant growth promotion and disease suppression can be recruited with three, consecutive 160-daycycles in the growth chamber. This rapid development of suppressiveness has previously been observed in greenhouse experiments (Lucas et al., 1993; Yin et al., 2013). If wheat genotypes can be identified that can speed the process it will be valuable since it takes years to naturally develop in no-till cropping systems. Progression of suppressiveness against *R. solani* AG-8 took five to ten years in Avon, South Australia (Roget, 1995), while it took eight to eleven years of no-till wheat monoculture in Ritzville, WA (Schillinger and Paulitz, 2014). If this progression could be enhanced by use of specific wheat genotypes, these varieties could be particularly important in transitioning to sustainable disease management systems, such as those involving reduced tillage. Our study identified genotype-specific microbiomes that are correlated with better plant growth and reduced root disease caused by *R. solani* AG-8. Differential abundance of Burkholderiales OTUs, specifically the genus *Janthinobacterium* in PI561727 and PI561725 cultivated soils was associated with reduced root disease and better growth. This same genus was recently reported by Yin et al. (2021) to exhibit antagonism against *R. solani* AG-8 from disease-suppressive soil. Thus, it can be inferred that these Burkholderiales OTUs could be a putative biological control agent against *R. solani* AG-8 as it has been associated with disease-suppressive soils (Mendes et al., 2011; Carrión et al., 2018) and that they can be recruited by specific wheat genotypes. These results were different from the wheat cycling experiments performed by Mazzola and Gu (2002), where suppression of *R. solani* AG-5 and AG-8 were associated with the differences in the composition of fluorescent pseudomonad population in orchard soils. However, taken together, wheat genotypes have the capability to recruit different bacterial taxa responsible for better plant growth and disease suppression, in a given soil type and agroecosystem. With this, use of specific wheat genotypes to recruit suppressive microbiome holds promise in furthering efforts to manipulate rhizosphere microbiomes to manage root rot disease caused by *R. solani* AG-8.

## Conclusion

In this study, wheat genotype and physiological stage shaped the microbiome, which was able to significantly alter soil suppression of *R. solani* AG-8. Longer growth cycles resulted in stronger genotype-specific recruitment of the microbiome and reduced the number of edges in ecological networks. Despite differences between the microbiomes associated with field- and growth chamber-grown plants, the conclusions remain that genotype-specific rhizosphere recruitment may be observed in both systems. This is fundamental to our approach in future studies examining the phenomenon of developing suppressive soils in shorter time periods. Furthermore, the wheat genotype-specific recruitment of particular bacterial taxa correlated with better plant growth in *R. solani* AG-8 inoculated soils and reduced root disease, which demonstrates that disease-suppressive soils can be attained with fewer growth cycles. Thus, using the appropriate wheat genotype to manipulate the rhizosphere microbiome could provide a sustainable approach to manage soil-borne disease. However, further validation is needed to strengthen the importance of taxa associated with these significant OTUs in soil disease suppression.

## Data Availability Statement

The raw Illumina sequencing data generated from the experiments is publicly available at the National Center for Biotechnology Information (NCBI) under the BioProject accession PRJNA734707. Metadata is shown in Supplementary Table 9.

## Author Contributions

CD-E prepared the experimental design, execution of the experiment, rhizosphere soil collection, DNA extraction, data analysis, and writing of the manuscript. RL assisted with the experimental design, data analysis, and writing of the manuscript. TS assisted in creation of the research project, in the design of experiments, and edited the manuscript. SH created the research project, assisted in the design and execution of experiments, and edited the manuscript. All authors contributed to the article and approved the submitted version.

## Funding

This work was supported by the Hatch project 1016563 and 1014527, the Washington Grain Commission, and the WSU Emerging Research Issues grant. Supplementary research funds were also given by the Harry E. Goldsworthy Wheat Research Fund.

## Conflict of Interest

The authors declare that the research was conducted in the absence of any commercial or financial relationships that could be construed as a potential conflict of interest.

## Publisher’s Note

All claims expressed in this article are solely those of the authors and do not necessarily represent those of their affiliated organizations, or those of the publisher, the editors and the reviewers. Any product that may be evaluated in this article, or claim that may be made by its manufacturer, is not guaranteed or endorsed by the publisher.

## Footnotes

^1^ https://briatte.github.io/ggnet/

## References

[ref1] Albanese D. Fontana P. De Filippo C. Cavalieri D. Donati C. (2015). MICCA: a complete and accurate software for taxonomic profiling of metagenomic data. Sci. Rep. 5:9743. doi: 10.1038/srep09743 25988396 PMC4649890

[ref2] Anderson M. J. Willis T. J. (2003). Canonical analysis of principal coordinates: a useful method of constrained ordination for ecology. Ecology 84, 511–525. doi: 10.1890/0012-9658(2003)084[0511:CAOPCA]2.0.CO;2

[ref3] Benítez M.-S. Gardener B. B. M. (2009). Linking sequence to function in soil bacteria: sequence-directed isolation of novel bacteria contributing to soilborne plant disease suppression. Appl. Environ. Microbiol. 75, 915–924. doi: 10.1128/AEM.01296-08, PMID: 19088312 PMC2643568

[ref4] Berendsen R. L. Pieterse C. M. J. Bakker P. A. H. M. (2012). The rhizosphere microbiome and plant health. Trends Plant Sci. 17, 478–486. doi: 10.1016/j.tplants.2012.04.001, PMID: 22564542

[ref5] Bulgarelli D. Garrido-Oter R. Münch P. C. Weiman A. Dröge J. Pan Y.. (2015). Structure and function of the bacterial root microbiota in wild and domesticated barley. Cell Host Microbe 17, 392–403. doi: 10.1016/j.chom.2015.01.011, PMID: 25732064 PMC4362959

[ref6] Carrión V. J. Cordovez V. Tyc O. Etalo D. W. de Bruijn I. de Jager V. C. L.. (2018). Involvement of Burkholderiaceae and sulfurous volatiles in disease-suppressive soils. ISME J. 12, 2307–2321. doi: 10.1038/s41396-018-0186-x, PMID: 29899517 PMC6092406

[ref7] Carver B. F. Whitmore W. E. Smith E. L. Bona L. (1993). Registration of four aluminum-tolerant winter wheat germplasms and two susceptible near-isolines. Crop Sci. 33, 1113–1114. doi: 10.2135/cropsci1993.0011183X003300050060x

[ref8] Chambers J. M. (1992). “Linear models,” in Statistical Models in S. eds. Chambers J. M. Hastie T. J. (Pacific Grove, CA: Wadsworth and Brooks/Cole), 95–144.

[ref9] Chaparro J. M. Badri D. V. Vivanco J. M. (2014). Rhizosphere microbiome assemblage is affected by plant development. ISME J. 8, 790–803. doi: 10.1038/ismej.2013.196, PMID: 24196324 PMC3960538

[ref10] Compant S. Clément C. Sessitsch A. (2010). Plant growth-promoting bacteria in the rhizo- and endosphere of plants: their role, colonization, mechanisms involved and prospects for utilization. Soil Biol. Biochem. 42, 669–678. doi: 10.1016/j.soilbio.2009.11.024

[ref11] Conover W. J. (1999). Practical Nonparametric Statistical. 3rd *Edn*. New York: John Wiley & Sons Inc.

[ref12] de Garcia Salamone I. E. Hynes R. K. Nelson L. M. (2005). “Role of cytokinins in plant growth promotion by rhizosphere bacteria,” in PGPR: Biocontrol and Biofertilization. ed. Siddiqui Z. A. (Dordrecht, Netherlands: Springer), 173–195.

[ref13] de Mendiburu F. (2009). Una herramienta de analisis estadistico para la investigacion agricola. master’s thesis. Rímac, Peru: Universidad Nacional de Ingenieria (UNI-PERU).

[ref14] Delhaize E. Ryan P. R. Randall P. J. (1993). Aluminum tolerance in wheat (*Triticum aestivum* L.) II. Aluminum-stimulated excretion of malic acid from root apices. Plant Physiol. 103, 695–702. doi: 10.1104/pp.103.3.695, PMID: 12231973 PMC159038

[ref15] Dennis P. G. Miller A. J. Hirsch P. R. (2010). Are root exudates more important than other sources of rhizodeposits in structuring rhizosphere bacterial communities? FEMS Microbiol. Ecol. 72, 313–327. doi: 10.1111/j.1574-6941.2010.00860.x 20370828

[ref16] Dias M. P. Bastos M. S. Xavier V. B. Cassel E. Astarita L. V. Santarém E. R. (2017). Plant growth and resistance promoted by *Streptomyces* spp. in tomato. Plant Physiol. Biochem. 118, 479–493. doi: 10.1016/j.pla-phy.2017.07.017 28756346

[ref17] Donaldson N. C. (1980). Soil Survey of Whitman County, Washington. Washington: Department of Agriculture, Soil Conservation Service.

[ref18] Doulcier G. Stouffer D. (2015). Rnetcarto: fast network modularity and roles computation by simulated annealing. R package version 0.2.4.

[ref19] Edwards J. Johnson C. Santos-Medellín C. Lurie E. Podishetty N. K. Bhatnagar S.. (2015). Structure, variation, and assembly of the root-associated microbiomes of rice. Proc. Natl. Acad. Sci. 112, E911–E920. doi: 10.1073/pnas.1414592112, PMID: 25605935 PMC4345613

[ref21] Fernandez A. Huang S. Seston S. Xing J. Hickey R. Criddle C.. (1999). How stable is stable? Function versus community composition. Appl. Environ. Microbiol. 65, 3697–3704. doi: 10.1128/AEM.65.8.3697-3704.1999, PMID: 10427068 PMC91553

[ref22] Fox J. Weisberg S. (2011). An R Companion to Applied Regression. 2nd *Edn*. California: SAGE Publications, Inc.

[ref23] Glick B. R. Cheng Z. Czarny J. Duan J. (2007). Promotion of plant growth by ACC deaminase-producing soil bacteria. Eur. J. Plant Pathol. 119, 329–339. doi: 10.1007/s10658-007-9162-4

[ref24] Goldstein A. H. (1986). Bacterial solubilization of mineral phosphates: historical perspective and future prospects. Am. J. Altern. Agri. 1, 51–57. doi: 10.1017/S0889189300000886

[ref25] Guimerà R. Amaral L. A. N. (2005). Functional cartography of complex metabolic networks. Nature 433, 895–900. doi: 10.1038/nature03288, PMID: 15729348 PMC2175124

[ref26] Hinsinger P. Bengough A. G. Vetterlein D. Young I. M. (2009). Rhizosphere: biophysics, biogeochemistry and ecological relevance. Plant Soil 321, 117–152. doi: 10.1007/s11104-008-9885-9

[ref27] Houde M. Diallo A. O. (2008). Identification of genes and pathways associated with aluminum stress and tolerance using transcriptome profiling of wheat near-isogenic lines. BMC Genomics 9:400. doi: 10.1186/1471-2164-9-400, PMID: 18752686 PMC2551624

[ref28] Human Microbiome Project Consortium (2012). Structure, function, and diversity of the healthy human microbiome. Nature 486, 207–214. doi: 10.1038/nature11234, PMID: 22699609 PMC3564958

[ref29] Inceoğlu O. Salles J. F. van Overbeek L. van Elsas J. D. (2010). Effects of plant genotype and growth stage on the betaproteobacterial communities associated with different potato cultivars in two fields. Appl. Environ. Microbiol. 76, 3675–3684. doi: 10.1128/AEM.00040-10, PMID: 20363788 PMC2876460

[ref30] Kelly B. J. Gross R. Bittinger K. Sherrill-Mix S. Lewis J. D. Collman R. G.. (2015). Power and sample-size estimation for microbiome studies using pairwise distances and PERMANOVA. Bioinformatics 31, 2461–2468. doi: 10.1093/bioinformatics/btv183, PMID: 25819674 PMC4514928

[ref31] Kim D. S. Cook R. J. Weller D. M. (1997). *Bacillus* sp. L324-92 for biological control of three root diseases of wheat grown with reduced tillage. Phytopathology 87, 551–558. doi: 10.1094/PHYTO.1997.87.5.551, PMID: 18945111

[ref32] Kruskal W. H. Wallis W. A. (1952). Use of ranks in one-criterion variance analysis. J. Am. Stat. Assoc. 47, 583–621.

[ref33] Kumar P. S. Brooker M. R. Dowd S. E. Camerlengo T. (2011). Target region selection is a critical determinant of community fingerprints generated by 16S pyrosequencing. PLoS One 6:e20956. doi: 10.1371/journal.pone.0020956, PMID: 21738596 PMC3126800

[ref34] Kurtz Z. D. Müller C. L. Miraldi E. R. Littman D. R. Blaser M. J. Bonneau R. A. (2015). Sparse and compositionally robust inference of microbial ecological networks. PLoS Comput. Biol. 11:e1004226. doi: 10.1371/journal.pcbi.1004226, PMID: 25950956 PMC4423992

[ref35] Kuzyakov Y. Domanski G. (2000). Carbon input by plants into the soil. Review. *J. Plant Nutr. Soil Sci.* 163, 421–431. doi: 10.1002/1522-2624(200008)163:4<421::AID-JPLN421>3.0.CO;2-R

[ref36] Lane D. J. (1991). “16S/23S rRNA sequencing” in Nucleic Acid Techniques in Bacterial Systematics. eds. Stackebrandt E. Goodfellow M. (New York, NY: John Wiley and Sons), 115–175.

[ref37] Legendre P. Legendre L. (2012). Numerical Ecology. 3rd *Edn*. Amsterdam: Elsevier.

[ref38] Levene H. (1960). “Robust tests for equality of variance” in Contributions to Probability and Statistics: Essays in Honor of Harold Hotelling. ed. Olkin I. (Palo Alto, CA: Stanford University Press), 278–292.

[ref39] Li J. Luo Z. Zhang C. Qu X. Chen M. Song T.. (2020). Seasonal variation in the rhizosphere and non-rhizosphere microbial community structures and functions of *camellia yuhsienensis* Hu. Microorganisms 8:1385. doi: 10.3390/microorganisms8091385, PMID: 32927703 PMC7564921

[ref40] Louca S. Jacques S. M. S. Pires A. P. F. Leal J. S. Srivastava D. S. Parfrey L. W.. (2016). High taxonomic variability despite stable functional structure across microbial communities. Nat. Ecol. Evol. 1:0015. doi: 10.1038/s41559-016-0015 28812567

[ref41] Love M. I. Huber W. Anders S. (2014). Moderated estimation of fold change and dispersion for RNA-seq data with DESeq2. Genome Biol. 15:550. doi: 10.1186/s13059-014-0550-8 25516281 PMC4302049

[ref42] Lucas P. Smiley R. W. Collins H. P. (1993). Decline of Rhizoctonia root rot on wheat in soils infested with *Rhizoctonia solani* AG-8. Phytopathology 83, 260–265. doi: 10.1094/Phyto-83-260

[ref43] Mahoney A. K. Yin C. Hulbert S. H. (2017). Community structure, species variation, and potential functions of rhizosphere-associated bacteria of different winter wheat (*Triticum aestivum*) cultivars. Front. Plant Sci. 8:132. doi: 10.3389/fpls.2017.00132, PMID: 28243246 PMC5303725

[ref44] Mazzola M. Gu Y.-H. (2002). Wheat genotype-specific induction of soil microbial communities suppressive to disease incited by *Rhizoctonia solani* anastomosis group (AG)-5 and (AG)-8. Phytopathology 92, 1300–1307. doi: 10.1094/PHYTO.2002.92.12.1300, PMID: 18943884

[ref45] McDonald D. Clemente J. C. Kuczynski J. Rideout J. R. Stombaugh J. Wendel D.. (2012). The biological observation matrix (BIOM) format or: how I learned to stop worrying and love the ome-ome. GigaScience 1:7. doi: 10.1186/2047-217X-1-7, PMID: 23587224 PMC3626512

[ref46] McMurdie P. J. Holmes S. (2013). Phyloseq: An R package for reproducible interactive analysis and graphics of microbiome census data. PLoS One 8:e61217. doi: 10.1371/journal.pone.0061217 23630581 PMC3632530

[ref47] Meinshausen N. Bühlmann P. (2006). High-dimensional graphs and variable selection with the lasso. Ann. Stat. 34, 1436–1462. doi: 10.1214/009053606000000281

[ref48] Mendes R. Kruijt M. de Bruijn I. Dekkers E. van der Voort M. Schneider J. H.. (2011). Deciphering the rhizosphere microbiome for disease-suppressive bacteria. Science 332, 1097–1100. doi: 10.1126/science.1203980, PMID: 21551032

[ref49] Micallef S. A. Channer S. Shiaris M. P. Colón-Carmona A. (2009). Plant age and genotype impact the progression of bacterial community succession in the Arabidopsis rhizosphere. Plant Signal. Behav. 4, 777–780. doi: 10.1093/jxb/erp053 19820328 PMC2801398

[ref50] Morais M. C. Mucha Â. Ferreira H. Gonçalves B. Bacelar E. Marques G. (2019). Comparative study of plant growth-promoting bacteria on the physiology, growth, and fruit quality of strawberry. J. Sci. Food Agric. 99, 5341–5349. doi: 10.1002/jsfa.9773, PMID: 31058322

[ref51] Okubara P. A. Schroeder K. L. Abatzoglou J. T. Paulitz T. C. (2014). Agroecological factors correlated to soil DNA concentrations of Rhizoctonia in dryland wheat production zones of Washington state, USA. Phytopathology 104, 683–691. doi: 10.1094/PHYTO-09-13-0269-R, PMID: 24915426

[ref52] Olesen J. M. Bascompte J. Dupont Y. L. Jordano P. (2007). The modularity of pollination networks. Proc. Natl. Acad. Sci. 104, 19891–19896. doi: 10.1073/pnas.0706375104 18056808 PMC2148393

[ref54] Paulitz T. C. Schroeder K. L. (2005). A new method for the quantification of *Rhizoctonia solani* and *R. oryzae* from soil. Plant Dis. 89, 767–772. doi: 10.1094/PD-89-0767, PMID: 30791249

[ref55] Peiffer J. A. Spor A. Koren O. Jin Z. Tringe S. G. Dangl J. L.. (2013). Diversity and heritability of the maize rhizosphere microbiome under field conditions. Proc. Natl. Acad. Sci. 110, 6548–6553. doi: 10.1073/pnas.1302837110 23576752 PMC3631645

[ref56] Pérez-Jaramillo J. E. Carrión V. J. Bosse M. Ferrão L. F. V. de Hollander M. Garcia A. A.. (2017). Linking rhizosphere microbiome composition of wild and domesticated *Phaseolus vulgaris* to genotypic and root phenotypic traits. ISME J. 11, 2244–2257. doi: 10.1038/ismej.2017.85, PMID: 28585939 PMC5607367

[ref57] Philippot L. Raaijmakers J. M. Lemanceau P. van der Putten W. H. (2013). Going back to the roots: the microbial ecology of the rhizosphere. Nat. Rev. Microbiol. 11, 789–799. doi: 10.1038/nrmicro3109, PMID: 24056930

[ref58] Pollak S. Cordero O. X. (2020). Microbiome shields plants from infection. Nat. Microbiol. 5, 978–979. doi: 10.1038/s41564-020-0766-1, PMID: 32710094

[ref59] Poudel R. Jumpponen A. Schlatter D. Paulitz T. C. McSpadden Gardener B. Kinkel L. L.. (2016). Microbiome networks: a systems framework for identifying candidate microbial assemblages for disease management. Phytopathology 106, 1083–1096. doi: 10.1094/PHYTO-02-16-0058-FI, PMID: 27482625

[ref60] Pumphrey F. V. Wilkins D. E. Hane D. C. Smiley R. W. (1987). Influence of tillage and nitrogen fertilizer on Rhizoctonia root rot (bare patch) of winter wheat. Plant Dis. 71, 125–127. doi: 10.1094/PD-71-0125

[ref61] Qiao Q. Wang F. Zhang J. Chen Y. Zhang C. Liu G.. (2017). The variation in the rhizosphere microbiome of cotton with soil type, genotype, and developmental stage. Sci. Rep. 7:3940. doi: 10.1038/s41598-017-04213-7 28638057 PMC5479781

[ref62] R Core Team (2017). R: A language and environment for statistical computing. R Foundation for Statistical Computing, Vienna, Austria. https://www.r-project.org/ (Accessed March 6, 2017).

[ref63] Rodríguez H. Fraga R. (1999). Phosphate solubilizing bacteria and their role in plant growth promotion. Biotechnol. Adv. 17, 319–339. doi: 10.1016/S0734-9750(99)00014-2, PMID: 14538133

[ref64] Rodríguez H. Goire I. Rodríguez M. (1996). Caracterización de cepas de Pseudomonas solubilizadoras de fósforo. Rev ICIDCA 30, 47–54.

[ref65] Roget D. K. (1995). Decline of root rot (*Rhizoctonia solani* AG-8) in wheat in a tillage and rotation experiment at Avon. South Australia. *Aust. J. of Expt. Agric.* 35, 1009–1013. doi: 10.1071/EA9951009

[ref66] Saleem M. Law A. D. Sahib M. R. Pervaiz Z. H. Zhang Q. (2018). Impact of root system architecture on rhizosphere and root microbiome. Rhizosphere 6, 47–51. doi: 10.1016/j.rhisph.2018.02.003

[ref67] Sasaki T. Yamamoto Y. Ezaki B. Katsuhara M. Ahn S. J. Ryan P. R.. (2004). A wheat gene encoding an aluminum-activated malate transporter. Plant J. 37, 645–653. doi: 10.1111/j.1365-313x.2003.01991.x, PMID: 14871306

[ref68] Scagliola M. Pii Y. Mimmo T. Cesco S. Ricciuti P. Crecchio C. (2016). Characterization of plant growth promoting traits of bacterial isolates from the rhizosphere of barley (*Hordeum vulgare* L.) and tomato (*Solanum lycopersicon* L.) grown under Fe sufficiency and deficiency. Plant Physiol. Biochem. 107, 187–196. doi: 10.1016/j.plaphy.2016.06.002 27295343

[ref69] Schillinger W. F. Paulitz T. C. (2014). Natural suppression of Rhizoctonia bare patch in a long-term no-till cropping systems experiment. Plant Dis. 98, 389–394. doi: 10.1094/PDIS-04-13-0420-RE, PMID: 30708450

[ref70] Schlemper T. R. Leite M. F. A. Lucheta A. R. Shimels M. Bouwmeester H. J. van Veen J. A.. (2017). Rhizobacterial community structure differences among sorghum cultivars in different growth stages and soils. FEMS Microbiol. Ecol. 93:fix096. doi: 10.1093/femsec/fix096, PMID: 28830071

[ref71] Shapiro S. S. Wilk M. B. (1965). An analysis of variance test for normality (complete samples). Biometrika 52, 591–611. doi: 10.1093/biomet/52.3-4.591

[ref72] Shi S. Nuccio E. Herman D. J. Rijkers R. Estera K. Li J.. (2015). Successional trajectories of rhizosphere bacterial communities over consecutive seasons. MBio 6:e00746-15. doi: 10.1128/mBio.00746-15 26242625 PMC4526712

[ref73] Smalla K. Wieland G. Buchner A. Zock A. Parzy J. Kaiser S.. (2001). Bulk and rhizosphere soil bacterial communities studied by denaturing gradient gel electrophoresis: plant-dependent enrichment and seasonal shifts revealed. Appl. Environ. Microbiol. 67, 4742–4751. doi: 10.1128/AEM.67.10.4742-4751.2001, PMID: 11571180 PMC93227

[ref74] Vurukonda S. S. K. P. Giovanardi D. Stefani E. (2018). Plant growth promoting and biocontrol activity of *Streptomyces* spp. as endophytes. Int. J. Mol. Sci. 19:952. doi: 10.3390/ijms19040952 PMC5979581 29565834

[ref75] Walters W. A. Jin Z. Youngblut N. Wallace J. G. Sutter J. Zhang W.. (2018). Large-scale replicated field study of maize rhizosphere identifies heritable microbes. Proc. Natl. Acad. Sci. 115, 7368–7373. doi: 10.1073/pnas.1800918115, PMID: 29941552 PMC6048482

[ref76] Weller D. M. Cook R. J. MacNish G. Bassett E. N. Powelson R. L. Petersen R. R. (1986). Rhizoctonia root rot of small grains favored by reduced tillage in the Pacific northwest. Plant Dis. 70, 70–73. doi: 10.1094/PD-70-70

[ref77] Weller D. M. Raaijmakers J. M. Gardener B. B. Thomashow L. S. (2002). Microbial populations responsible for specific soil suppressiveness to plant pathogens. Annu. Rev. Phytopathol. 40, 309–348. doi: 10.1146/annurev.phyto.40.030402.110010, PMID: 12147763

[ref78] Wickham H. (2009). Ggplot 2: Elegant Graphics for Data Analysis. New York: Springer.

[ref79] Wilkinson G. N. Rogers C. E. (1973). Symbolic description of factorial models for analysis of variance. J. R. Stat. Soc. Ser. C. Appl. Stat. 22, 392–399.

[ref80] Yin C. Casas Vargas J. M. Schlatter D. C. Hagerty C. H. Hulbert S. H. Paulitz T. C. (2021). Rhizosphere community selection reveals bacteria associated with reduced root disease. Microbiome 9:86. doi: 10.1186/s40168-020-00997-5 33836842 PMC8035742

[ref81] Yin C. Hulbert S. H. Schroeder K. L. Mavrodi O. Mavrodi D. Dhingra A.. (2013). Role of bacterial communities in the natural suppression of *Rhizoctonia solani* bare patch disease of wheat (*Triticum aestivum* L.). Appl. Env. Microbiol. 79, 7428–7438. doi: 10.1128/AEM.01610-13, PMID: 24056471 PMC3837727

[ref82] Yuan J. Chaparro J. M. Manter D. K. Zhang R. Vivanco J. M. Shen Q. (2015). Roots from distinct plant developmental stages are capable of rapidly selecting their own microbiome without the influence of environmental and soil edaphic factors. Soil Biol. Biochem. 89, 206–209. doi: 10.1016/j.soilbio.2015.07.009

