# Supplementary material for: Wheat Genotype-Specific Recruitment of Rhizosphere Bacterial Microbiota Under Controlled Environments
Source: Front Plant Sci. 2021 Dec 1;12:718264. doi: 10.3389/fpls.2021.718264 (PMC8671755; doi:10.3389/fpls.2021.718264)
Supplement: Supplementary file 1 [file Data_Sheet_1.docx]

**Supplementary Table 1 :** Summary of alpha-diversity of the rhizosphere microbiome among different wheat genotypes.

| **Genotype** | **Shannon Index** | | | | |
| --- | --- | --- | --- | --- | --- |
|  | **P28^a^** | **P35a^b^** | **P35b^c^** | **P160^d^** | |
| Eltan | 3.75 ± 1.77 | 5.22 ± 0.48 | 5.76 ± 0.50 | 5.55 ± 0.20 | |
| Hill81 | 5.93 ± 0.27 | 5.49 ± 0.29 | 6.03 ± 0.53 | 5.59 ± 0.28 | |
| Lewjain | 5.64 ± 0.25 | 5.58 ± 0.24 | 6.10 ± 0.31 | 5.68 ± 0.13 | |
| Madsen | 4.60 ± 1.35 | 5.51 ± 0.26 | 5.96 ± 0.24 | 5.68 ± 0.27 | |
| PI561725 | 5.92 ± 0.22 | 5.65 ± 0.27 | 5.93 ± 0.47 | 5.29 ± 0.49 | |
| PI561727 | 4.980 ± 1.41 | 5.42 ± 0.30 | 5.75 ± 0.42 | 5.28 ± 0.32 | |
| ^a^ Three 28-day cycles | | | | | |
| ^b^ Three 35-day cycles (Trial 1) | | | | | |
| ^c^ Three 35-day cycles (Trial 2) | | | | | |
| ^d^ Three 160-day cycles | | | | | |


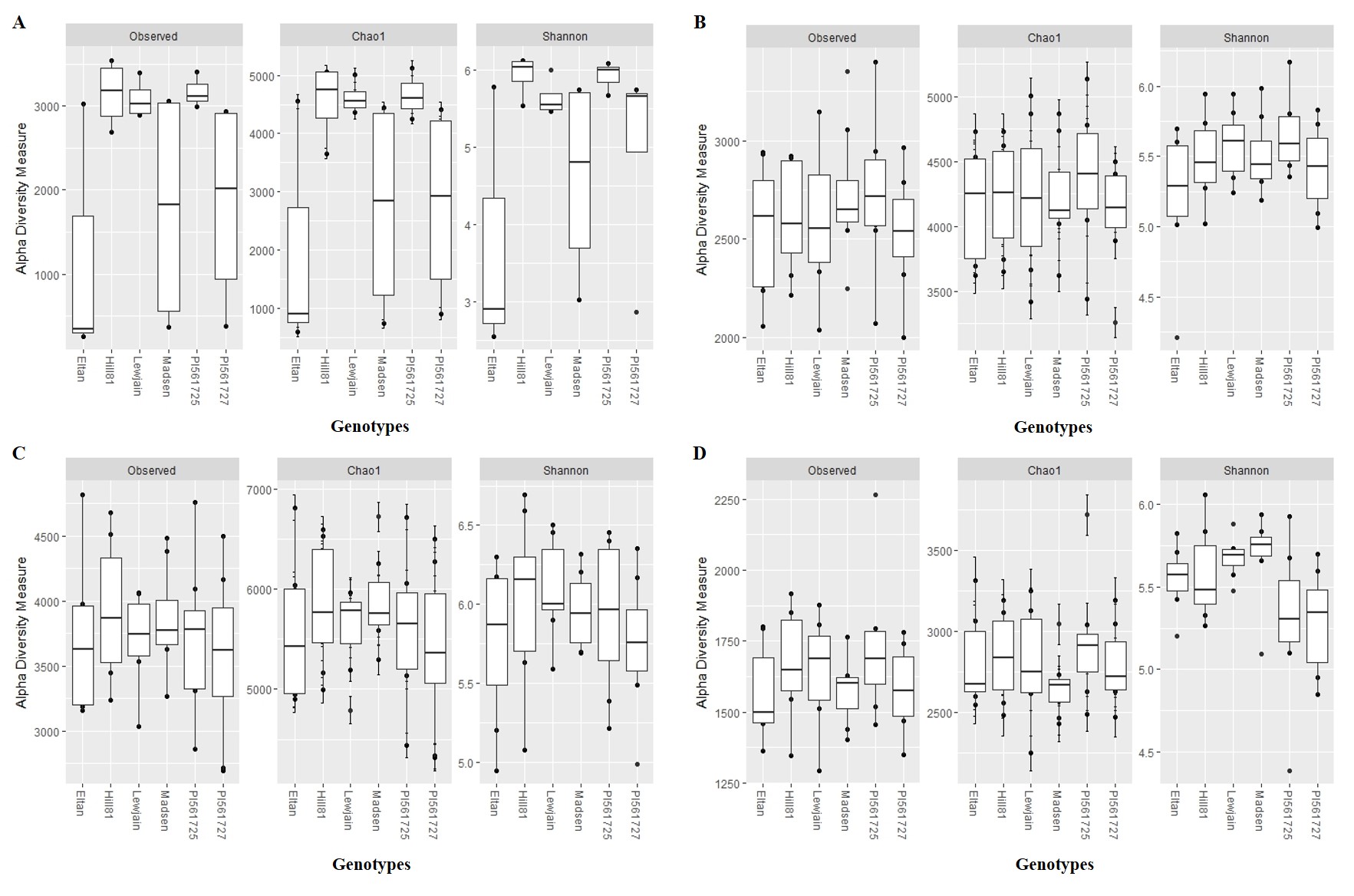


**Supplementary Figure 1 :** **Alpha diversity of the rhizosphere microbiome of six winter wheat genotypes. (A-D)** 28-, 35- (Trial 1), 35- (Trial 2), and 160-day cycle.

**Supplementary Table 2 :** PERMANOVA pair-wise tests between the rhizosphere microbiome of six winter wheat genotypes and across experiments based on Bray-Curtis similarity (P-value at 999 permutations).

| a. 35-Day Cycles Trial 1 (P35a) | | |  |  |  |
| --- | --- | --- | --- | --- | --- |
| Genotype | Eltan | Hill81 | Lewjain | Madsen | PI561725 |
| Hill81 | 0.088 |  |  |  |  |
| Lewjain | 0.038 | 0.456 |  |  |  |
| Madsen | 0.090 | 0.262 | 0.333 |  |  |
| PI561725 | 0.066 | 0.160 | 0.306 | 0.143 |  |
| PI561727 | 0.067 | 0.145 | 0.158 | 0.516 | 0.279 |
|  |  |  |  |  |  |
| b. 35-Day Cycles Trial 2 (P35b) | | |  |  |  |
| Genotype | Eltan | Hill81 | Lewjain | Madsen | PI561725 |
| Hill81 | 0.039 |  |  |  |  |
| Lewjain | 0.052 | 0.092 |  |  |  |
| Madsen | 0.081 | 0.286 | 0.191 |  |  |
| PI561725 | 0.067 | 0.242 | 0.150 | 0.269 |  |
| PI561727 | 0.169 | 0.165 | 0.064 | 0.317 | 0.300 |
|  |  |  |  |  |  |
| c. 160-day Cycles (P160) | |  |  |  |  |
| Genotype | Eltan | Hill81 | Lewjain | Madsen | PI561725 |
| Hill81 | 0.620 |  |  |  |  |
| Lewjain | 0.120 | 0.248 |  |  |  |
| Madsen | 0.091 | 0.384 | 0.120 |  |  |
| PI561725 | 0.013 | 0.011 | 0.013 | 0.006 |  |
| PI561727 | 0.030 | 0.030 | 0.009 | 0.011 | 0.400 |
|  |  |  |  |  |  |
| d. Growth chamber and field experiment | | | |  |  |
| Experiments | Field | P28 | P35a | P35b |  |
| P28 | 0.001 |  |  |  |  |
| P35a | 0.001 | 0.001 |  |  |  |
| P35b | 0.001 | 0.001 | 0.001 |  |  |
| P160 | 0.001 | 0.001 | 0.001 | 0.001 |  |
|  |  |  |  |  |  |

**Supplementary Table 3 :** Bray-Curtis similarity between the rhizosphere microbiome of six winter wheat genotypes and across experiments.

| a. 35-Day Cycles Trial 1 (P35a) | | |  |  |  |  |
| --- | --- | --- | --- | --- | --- | --- |
| Genotype | Eltan | Hill81 | Lewjain | Madsen | PI561725 | PI561727 |
| Hill81 | 66.97 |  |  |  |  |  |
| Lewjain | 68.00 | 70.08 |  |  |  |  |
| Madsen | 67.47 | 69.24 | 70.13 |  |  |  |
| PI561725 | 68.23 | 69.34 | 70.54 | 69.43 |  |  |
| PI561727 | 68.28 | 69.25 | 70.29 | 70.34 | 70.47 | 70.74 |
|  |  |  |  |  |  |  |
| b. 35-Day Cycles Trial 2 (P35b) | | |  |  |  |  |
| Genotype | Eltan | Hill81 | Lewjain | Madsen | PI561725 | PI561727 |
| Hill81 | 64.74 |  |  |  |  |  |
| Lewjain | 65.46 | 65.40 |  |  |  |  |
| Madsen | 67.52 | 67.59 | 67.91 |  |  |  |
| PI561725 | 66.38 | 66.71 | 66.92 | 69.10 |  |  |
| PI561727 | 66.43 | 65.87 | 65.58 | 68.63 | 67.56 | 66.49 |
|  |  |  |  |  |  |  |
| c. 160-day Cycles (P160) | |  |  |  |  |  |
| Genotype | Eltan | Hill81 | Lewjain | Madsen | PI561725 | PI561727 |
| Hill81 | 65.61 |  |  |  |  |  |
| Lewjain | 64.15 | 64.30 |  |  |  |  |
| Madsen | 66.00 | 66.77 | 66.39 |  |  |  |
| PI561725 | 60.28 | 60.19 | 57.31 | 59.72 |  |  |
| PI561727 | 61.91 | 61.73 | 58.07 | 61.18 | 65.71 | 65.11 |
|  |  |  |  |  |  |  |
| d. Growth chamber and field experiment | | | |  |  |  |
| Experiments | Field | P28 | P35a | P35b |  |  |
| P28 | 31.79 |  |  |  |  |  |
| P35a | 33.01 | 41.16 |  |  |  |  |
| P35b | 33.45 | 38.52 | 42.49 |  |  |  |
| P160 | 31.92 | 39.02 | 41.83 | 41.24 |  |  |
|  |  |  |  |  |  |  |
|  |  |  |  |  |  |  |


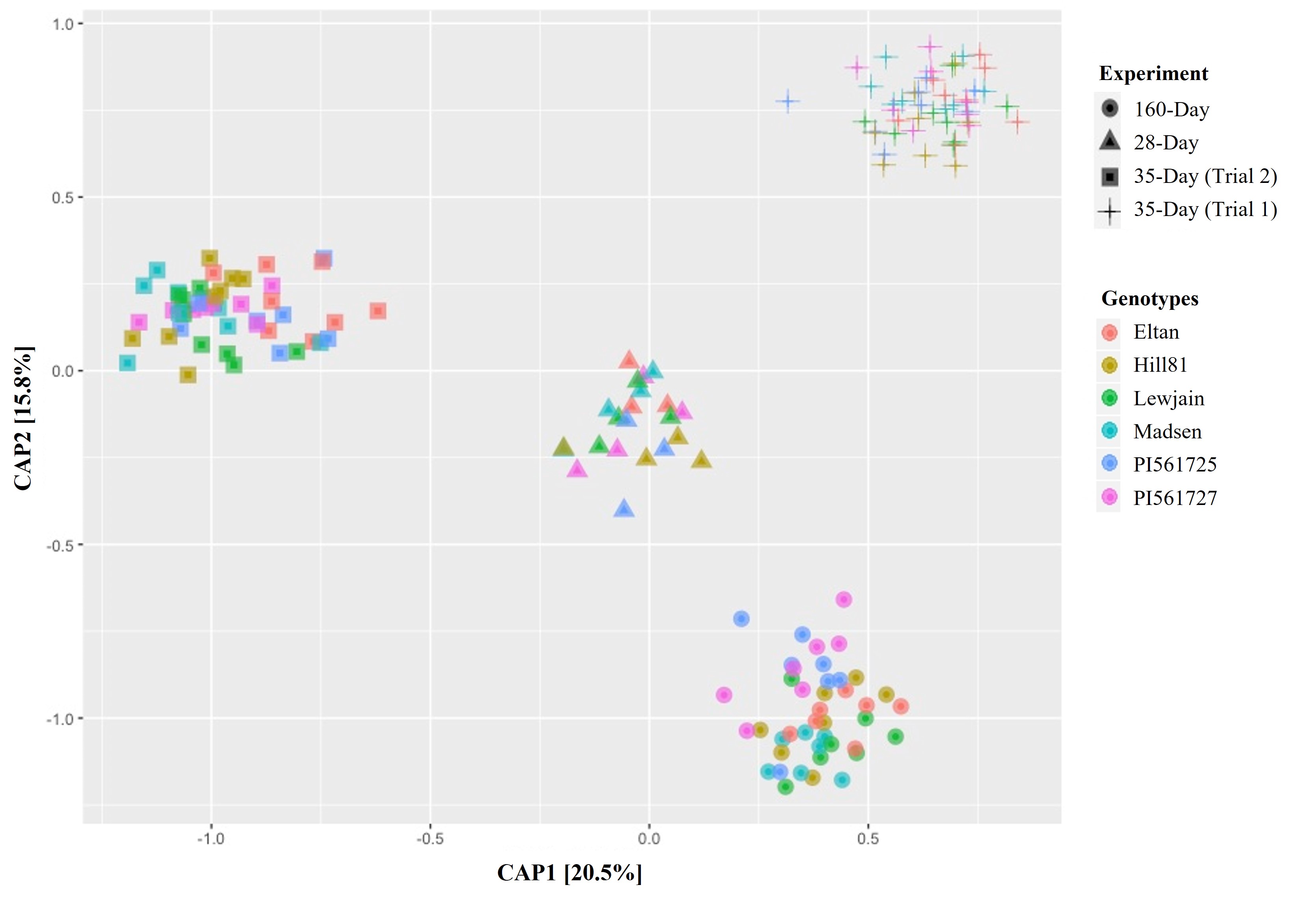


**Supplementary Figure 2: CAP plot of Bray-Curtis distances among bacterial communities of six winter wheat genotypes grown in Pullman soil across different growth chamber cycling lengths.** Different shapes represent different cycling experiments while different wheat genotypes are represented by different colors.


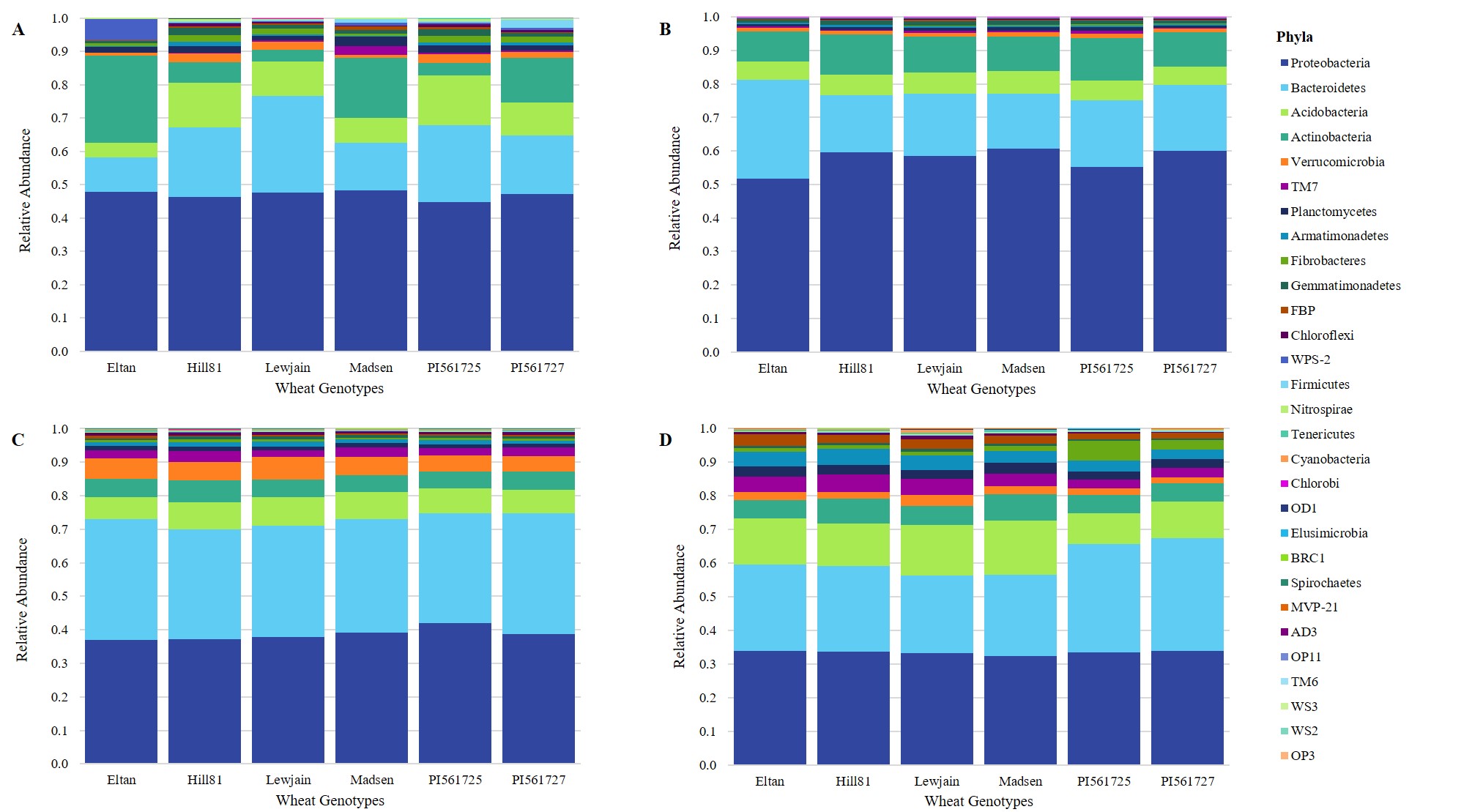


**Supplementary Figure 3:** **Most abundant bacterial taxa of six winter wheat genotypes grown in Pullman soil under different growth chamber cycling lengths. (A-D)** 28-, 35- (Trial 1), 35- (Trial 2), 160-day cycle. Most abundant bacterial phyla after filtering out OTUs with <0.001 relative abundance.

**Supplementary Table 4 :** Percent abundance of phyla of the most abundant bacterial taxa in the six winter wheat varieties among growth chamber experiment.

| **Dataset** | **Phyla*** | **Eltan** | **Hill81** | **Lewjain** | **Madsen** | **PI561725** | **PI561727** | **Rank** |
| --- | --- | --- | --- | --- | --- | --- | --- | --- |
| P28 | Proteobacteria | 47.8 | 46.3 | 47.6 | 48.4 | 44.9 | 47.1 | 1 |
| P28 | Bacteroidetes | 10.3 | 20.8 | 29.0 | 14.3 | 23.1 | 17.7 | 2 |
| P28 | Actinobacteria | 26.1 | 6.0 | 3.4 | 18.0 | 3.8 | 13.5 | 3 |
| P28 | Acidobacteria | 4.4 | 13.6 | 10.4 | 7.4 | 14.9 | 9.8 | 4 |
| P28 | Planctomycetes | 1.7 | 2.1 | 1.3 | 2.9 | 2.2 | 1.6 | 5 |
| P28 | Verrucomicrobia | 0.8 | 2.6 | 2.5 | 0.9 | 2.5 | 1.9 | 6 |
| P28 | Fibrobacteres | 0.9 | 2.1 | 1.6 | 0.6 | 2.1 | 1.8 | 7 |
| P28 | WPS-2 | 6.3 | 0.4 | 0.2 | 0.9 | 0.4 | 0.6 | 8 |
| P28 | Gemmatimonadetes | 0.6 | 2.1 | 1.2 | 1.0 | 2.0 | 1.1 | 9 |
| P28 | Firmicutes | 0.1 | 0.4 | 0.4 | 1.1 | 0.5 | 2.6 | 10 |
| P28 | TM7 | 0.1 | 0.3 | 0.3 | 2.6 | 0.4 | 0.3 | 11 |
| P28 | Armatimonadetes | 0.2 | 1.1 | 0.6 | 0.4 | 0.9 | 0.8 | 12 |
| P28 | Chloroflexi | 0.1 | 0.8 | 0.4 | 0.2 | 0.8 | 0.7 | 13 |
| P28 | FBP | 0.2 | 0.5 | 0.4 | 1.1 | 0.5 | 0.2 | 14 |
| P28 | Nitrospirae | 0.1 | 0.5 | 0.3 | 0.2 | 0.6 | 0.3 | 15 |
| P28 | Chlorobi | 0.1 | 0.1 | 0.1 | 0.1 | 0.1 | 0.1 | 16 |
| P28 | Elusimicrobia | 0.0 | 0.1 | 0.0 | 0.0 | 0.1 | 0.0 | 17 |
| P28 | BRC1 | 0.0 | 0.0 | 0.0 | 0.0 | 0.1 | 0.0 | 18 |
| P28 | Cyanobacteria | 0.0 | 0.0 | 0.1 | 0.0 | 0.0 | 0.0 | 19 |
| P28 | OD1 | 0.0 | 0.0 | 0.0 | 0.0 | 0.0 | 0.0 | 20 |
| P28 | Tenericutes | 0.0 | 0.1 | 0.0 | 0.0 | 0.0 | 0.0 | 21 |
| P28 | AD3 | 0.0 | 0.0 | 0.0 | 0.0 | 0.0 | 0.0 | 22 |
| P35a | Proteobacteria | 51.7 | 59.5 | 58.4 | 60.7 | 55.1 | 60.0 | 1 |
| P35a | Bacteroidetes | 29.5 | 17.0 | 18.6 | 16.3 | 20.0 | 19.7 | 2 |
| P35a | Actinobacteria | 8.9 | 12.1 | 10.7 | 10.3 | 12.6 | 10.2 | 3 |
| P35a | Acidobacteria | 5.4 | 6.2 | 6.4 | 6.9 | 6.0 | 5.5 | 4 |
| P35a | Verrucomicrobia | 1.2 | 1.1 | 1.2 | 1.2 | 1.4 | 1.0 | 5 |
| P35a | Gemmatimonadetes | 0.9 | 1.3 | 1.4 | 1.3 | 1.1 | 0.9 | 6 |
| P35a | Planctomycetes | 0.7 | 0.9 | 0.9 | 1.2 | 1.1 | 0.7 | 7 |
| P35a | Armatimonadetes | 0.4 | 0.5 | 0.6 | 0.5 | 0.5 | 0.5 | 8 |
| P35a | TM7 | 0.3 | 0.3 | 0.5 | 0.4 | 0.7 | 0.3 | 9 |
| P35a | Chloroflexi | 0.3 | 0.3 | 0.3 | 0.3 | 0.4 | 0.3 | 10 |
| P35a | FBP | 0.2 | 0.2 | 0.4 | 0.3 | 0.2 | 0.3 | 11 |

Supplementary Table 4 continued…

| **Dataset** | **Phyla*** | **Eltan** | **Hill81** | **Lewjain** | **Madsen** | **PI561725** | **PI561727** | **Rank** |
| --- | --- | --- | --- | --- | --- | --- | --- | --- |
| P35a | Firmicutes | 0.1 | 0.2 | 0.2 | 0.2 | 0.2 | 0.2 | 12 |
| P35a | Fibrobacteres | 0.1 | 0.2 | 0.1 | 0.1 | 0.3 | 0.1 | 13 |
| P35a | Nitrospirae | 0.1 | 0.1 | 0.1 | 0.1 | 0.1 | 0.1 | 14 |
| P35a | WPS-2 | 0.1 | 0.1 | 0.1 | 0.1 | 0.1 | 0.1 | 15 |
| P35a | Chlorobi | 0.0 | 0.0 | 0.0 | 0.0 | 0.0 | 0.0 | 16 |
| P35a | Elusimicrobia | 0.0 | 0.0 | 0.0 | 0.0 | 0.0 | 0.0 | 17 |
| P35a | BRC1 | 0.0 | 0.0 | 0.0 | 0.0 | 0.0 | 0.0 | 18 |
| P35a | OD1 | 0.0 | 0.0 | 0.0 | 0.0 | 0.0 | 0.0 | 19 |
| P35a | Cyanobacteria | 0.0 | 0.0 | 0.0 | 0.0 | 0.0 | 0.0 | 20 |
| P35b | Proteobacteria | 37.0 | 37.2 | 37.9 | 39.2 | 42.0 | 38.7 | 1 |
| P35b | Bacteroidetes | 36.0 | 32.7 | 33.2 | 33.9 | 32.7 | 36.0 | 2 |
| P35b | Acidobacteria | 6.6 | 8.0 | 8.5 | 7.9 | 7.5 | 7.0 | 3 |
| P35b | Verrucomicrobia | 6.1 | 5.5 | 6.8 | 5.5 | 4.9 | 4.7 | 4 |
| P35b | Actinobacteria | 5.5 | 6.6 | 5.3 | 5.1 | 4.9 | 5.5 | 5 |
| P35b | TM7 | 2.3 | 3.3 | 1.9 | 2.7 | 2.1 | 2.5 | 6 |
| P35b | Armatimonadetes | 1.1 | 1.3 | 1.5 | 1.2 | 1.2 | 1.0 | 7 |
| P35b | Planctomycetes | 1.3 | 1.4 | 1.2 | 1.2 | 1.2 | 1.0 | 8 |
| P35b | Chloroflexi | 0.9 | 1.0 | 1.0 | 0.7 | 0.8 | 0.9 | 9 |
| P35b | Gemmatimonadetes | 0.5 | 0.8 | 0.8 | 0.7 | 0.7 | 0.6 | 10 |
| P35b | Fibrobacteres | 0.7 | 0.8 | 0.7 | 0.5 | 0.7 | 0.6 | 11 |
| P35b | FBP | 0.7 | 0.3 | 0.4 | 0.5 | 0.3 | 0.5 | 12 |
| P35b | Tenericutes | 0.7 | 0.4 | 0.3 | 0.2 | 0.4 | 0.4 | 13 |
| P35b | Cyanobacteria | 0.3 | 0.2 | 0.1 | 0.2 | 0.2 | 0.3 | 14 |
| P35b | Firmicutes | 0.1 | 0.1 | 0.3 | 0.1 | 0.1 | 0.1 | 15 |
| P35b | Nitrospirae | 0.1 | 0.1 | 0.1 | 0.1 | 0.1 | 0.1 | 16 |
| P35b | Spirochaetes | 0.0 | 0.1 | 0.1 | 0.0 | 0.0 | 0.0 | 17 |
| P35b | Chlorobi | 0.0 | 0.1 | 0.1 | 0.0 | 0.0 | 0.0 | 18 |
| P35b | WPS-2 | 0.0 | 0.0 | 0.0 | 0.0 | 0.0 | 0.0 | 19 |
| P35b | OD1 | 0.0 | 0.0 | 0.0 | 0.0 | 0.0 | 0.0 | 20 |
| P35b | Elusimicrobia | 0.0 | 0.0 | 0.0 | 0.0 | 0.0 | 0.0 | 21 |
| P35b | BRC1 | 0.0 | 0.0 | 0.0 | 0.0 | 0.0 | 0.0 | 22 |
| P160 | Proteobacteria | 34.0 | 33.7 | 33.2 | 32.4 | 33.4 | 33.9 | 1 |
| P160 | Bacteroidetes | 25.5 | 25.5 | 23.0 | 24.2 | 32.2 | 33.4 | 2 |
| P160 | Acidobacteria | 13.7 | 12.7 | 15.1 | 16.0 | 9.3 | 11.0 | 3 |

Supplementary Table 4 continued…

| **Dataset** | **Phyla*** | **Eltan** | **Hill81** | **Lewjain** | **Madsen** | **PI561725** | **PI561727** | **Rank** |
| --- | --- | --- | --- | --- | --- | --- | --- | --- |
| P160 | Actinobacteria | 5.5 | 7.3 | 5.8 | 7.9 | 5.4 | 5.4 | 4 |
| P160 | TM7 | 4.6 | 5.2 | 4.8 | 3.7 | 2.5 | 2.7 | 5 |
| P160 | Armatimonadetes | 4.4 | 4.7 | 4.2 | 3.5 | 3.1 | 2.9 | 6 |
| P160 | Planctomycetes | 3.0 | 2.9 | 2.7 | 3.2 | 2.6 | 2.6 | 7 |
| P160 | FBP | 3.5 | 2.4 | 3.0 | 2.2 | 1.7 | 1.6 | 8 |
| P160 | Verrucomicrobia | 2.4 | 2.0 | 3.2 | 2.5 | 1.9 | 1.8 | 9 |
| P160 | Fibrobacteres | 1.0 | 1.1 | 1.2 | 1.6 | 5.9 | 2.7 | 10 |
| P160 | Chloroflexi | 0.6 | 0.7 | 0.9 | 0.8 | 0.4 | 0.4 | 11 |
| P160 | Gemmatimonadetes | 0.6 | 0.6 | 0.7 | 0.7 | 0.5 | 0.5 | 12 |
| P160 | Tenericutes | 0.4 | 0.3 | 0.5 | 0.5 | 0.1 | 0.1 | 13 |
| P160 | Nitrospirae | 0.2 | 0.2 | 0.3 | 0.2 | 0.3 | 0.3 | 14 |
| P160 | Cyanobacteria | 0.1 | 0.2 | 0.8 | 0.1 | 0.1 | 0.1 | 15 |
| P160 | Firmicutes | 0.1 | 0.2 | 0.2 | 0.2 | 0.3 | 0.3 | 16 |
| P160 | OD1 | 0.1 | 0.1 | 0.1 | 0.1 | 0.2 | 0.1 | 17 |
| P160 | Chlorobi | 0.0 | 0.1 | 0.1 | 0.1 | 0.1 | 0.1 | 18 |
| P160 | MVP-21 | 0.0 | 0.0 | 0.1 | 0.1 | 0.0 | 0.0 | 19 |
| P160 | WPS-2 | 0.0 | 0.0 | 0.0 | 0.0 | 0.0 | 0.0 | 20 |
| P160 | Elusimicrobia | 0.0 | 0.0 | 0.0 | 0.0 | 0.0 | 0.0 | 21 |
| P160 | BRC1 | 0.0 | 0.0 | 0.0 | 0.0 | 0.0 | 0.0 | 22 |
| * Phyla of merged OTUs after filtering out OTUs with < 0.001 relative abundance | | | | | | | | |

**Supplementary Table 5 :** Top 50 differentially abundant OTUs across six winter wheat varieties in four growth chamber cycling experiments

| **Dataset** | **OTUs** | **Adj. P-Value*** | **Comparison** | **Family** | **Scientific Name** |
| --- | --- | --- | --- | --- | --- |
| P160 | DENOVO30 | 9.26E-03 | lower in PI561725 & PI561727 than Lewjain | A4b | *Unidentified* |
| P160 | DENOVO81 | 8.88E-03 | lower in PI561727 & PI561725 than Eltan, Lewjain, Madsen, Hill81 | Actinospicaceae | *Unidentified* |
| P160 | DENOVO121 | 9.19E-03 | lower in PI561727 & PI561725 than Madsen; lower in PI561725 than Hill81 | Actinosynnemataceae | *Unidentified* |
| P160 | REF6743 | 8.82E-04 | lower in PI561725 & PI561727 than Eltan, Lewjain, Madsen, Hill81 | Anaeroplasmataceae | *Asteroleplasma* sp*.* |
| P160 | DENOVO2959 | 6.84E-04 | lower in PI561727 & PI561725 than Eltan, Lewjain, Madsen, Hill81 | Anaeroplasmataceae | *Asteroleplasma* sp. |
| P160 | DENOVO2719 | 1.11E-02 | lower in PI561727 & PI561725 than Eltan, Lewjain, Hill81; lower in PI561725 than Madsen | Anaeroplasmataceae | *Asteroleplasma* sp. |
| P160 | REF4888 | 4.03E-03 | higher in PI561727 & PI561725 than Eltan, Lewjain, Madsen | Caulobacteraceae | *Caulobacter* sp. |
| P160 | REF416 | 4.65E-03 | lower in PI561725 & PI561727 than Lewjain, Madsen | Chitinophagaceae | *Unidentified* |
| P160 | REF3713 | 1.09E-02 | lower in PI561725 & PI561727 than Eltan, Lewjain, Madsen, Hill81 | Chitinophagaceae | *Unidentified* |
| P160 | REF3149 | 8.77E-03 | lower in PI561727 than Lewjain, Madsen | Chitinophagaceae | *Unidentified* |
| P160 | DENOVO2329 | 1.31E-02 | lower in PI561727 than Lewjain, Madsen | Chthoniobacteraceae | *Unidentified* |
| P160 | DENOVO125 | 1.39E-02 | lower in PI561725 than Eltan, Lewjain | Chthoniobacteraceae | *Chthoniobacter* sp. |
| P160 | REF4717 | 2.99E-03 | higher in PI561727 & PI561725 than Eltan, Lewjain, Madsen, Hill81 | Comamonadaceae | *Variovorax paradoxus* |
| P160 | REF1650 | 1.08E-02 | lower in PI561727 & PI561725 than Hill81, Lewjain, Madsen | Comamonadaceae | *Unidentified* |
| P160 | DENOVO123 | 1.25E-02 | lower in PI561727 & PI561725 than Lewjain | Cytophagaceae | *Unidentified* |

Supplementary Table 5 continued…

| **Dataset** | **OTUs** | **Adj. P-Value*** | **Comparison** | **Family** | **Scientific Name** |
| --- | --- | --- | --- | --- | --- |
| P160 | REF6894 | 1.39E-02 | higher in Lewjain than PI561725, PI561727; lower in PI561725 than Eltan, Hill81 | Ellin6075 | *Unidentified* |
| P160 | REF5534 | 2.75E-03 | higher in Lewjain than PI561725 | Ellin6075 | *Unidentified* |
| P160 | REF5326 | 5.47E-03 | higher in Lewjain than PI561725, PI561727 | Ellin6075 | *Unidentified* |
| P160 | REF5260 | 7.97E-04 | higher in Lewjain than PI561725, PI561727 | Ellin6075 | *Unidentified* |
| P160 | REF4374 | 1.33E-02 | lower in PI561727 & PI561725 than Lewjain; lower in PI561725 than Eltan, Madsen | Ellin6075 | *Unidentified* |
| P160 | REF3567 | 1.00E-02 | higher in Lewjain than PI561727, Eltan, Hill81, PI561725; higher in Madsen than PI561725, PI561727; lower in PI561725 than Eltan | Ellin6075 | *Unidentified* |
| P160 | DENOVO3101 | 4.16E-03 | lower in PI561725 than Eltan | Fimbriimonadaceae | *Fimbriimonas* sp. |
| P160 | REF1644 | 9.54E-03 | lower in Madsen than PI561725 | Hyphomicrobiaceae | *Rhodoplanes* sp. |
| P160 | REF3099 | 8.51E-03 | higher in PI561725 than Lewjain, Madsen, Hill81; higher in PI561727 & PI561725 than Eltan | Koribacteraceae | *Candidatus Koribacter* sp. |
| P160 | REF2862 | 9.54E-03 | higher in Madsen than PI561725 | Nocardioidaceae | *Kribbella* sp. |
| P160 | REF3578 | 3.25E-05 | higher in PI561725 & PI561727 than Eltan, Lewjain, Madsen, Hill81 | Oxalobacteraceae | *Janthinobacterium* sp. |
| P160 | REF7627 | 5.75E-03 | higher in Lewjain than PI561725, PI561727 | Pirellulaceae | *Unidentified* |
| P160 | REF4027 | 3.00E-03 | higher in PI561725 & PI561727 than Eltan, Lewjain, Madsen | Rhizobiaceae | *Unidentified* |
| P160 | REF8018 | 3.07E-03 | higher in PI561725 & PI561727 than Eltan, Lewjain, Madsen, Hill81 | Sphingobacteriaceae | *Unidentified* |
| P160 | REF603 | 8.22E-04 | higher in PI561725 & PI561727 than Eltan, Hill81, Lewjain, Madsen | Sphingobacteriaceae | *Unidentified* |
| P160 | REF3283 | 9.50E-03 | higher in PI561727 & PI561725 than Lewjain, Madsen, Eltan, Hill81 | Sphingobacteriaceae | *Unidentified* |

Supplementary Table 5 continued…

| **Dataset** | **OTUs** | **Adj. P-Value*** | **Comparison** | **Family** | **Scientific Name** |
| --- | --- | --- | --- | --- | --- |
| P160 | DENOVO4 | 1.29E-02 | lower in PI561725 than Eltan, Lewjain, Madsen; lower in PI561725 than Hill81 | Sphingobacteriaceae | *Unidentified* |
| P160 | DENOVO3719 | 8.13E-03 | lower in PI561725 than Eltan, Lewjain | Sphingobacteriaceae | *Unidentified* |
| P160 | DENOVO266 | 1.32E-02 | lower in PI561727 & PI561725 than Eltan, Madsen, Lewjain, Hill81 | Sphingobacteriaceae | *Unidentified* |
| P160 | DENOVO2423 | 1.05E-02 | higher in PI561725 than Eltan, Madsen, Lewjain, Hill81; higher in PI561727 than Eltan | Sphingobacteriaceae | *Unidentified* |
| P160 | DENOVO11 | 1.40E-02 | higher in PI561725 than Eltan, Hill81, Lewjain, Madsen; higher in PI561727 than Eltan, Lewjain | Sphingobacteriaceae | *Unidentified* |
| P160 | REF6706 | 6.05E-03 | higher in Lewjain than PI561725, PI561727 | Sphingomonadaceae | *Sphingomonas* sp. |
| P160 | REF2226 | 9.60E-03 | lower in PI561725 & PI561727 than Lewjain, Eltan, Madsen, Hill81 | Sphingomonadaceae | *Kaistobacter* sp. |
| P160 | REF2166 | 1.09E-02 | lower in PI561725 than Hill81, Lewjain, Madsen, Eltan | Sphingomonadaceae | *Sphingomonas wittichii* |
| P160 | REF12 | 6.48E-04 | higher in Lewjain than PI561725, Hill81, PI561727 | Sphingomonadaceae | *Kaistobacter* sp. |
| P160 | REF4774 | 1.31E-02 | higher in PI561725 than Eltan | Streptomycetaceae | *Streptomyces* sp. |
| P160 | REF4166 | 1.09E-02 | higher in PI561725 & PI561727 than Eltan, Lewjain, Madsen | Streptomycetaceae | *Streptomyces* sp. |
| P160 | REF961 | 6.81E-03 | higher in PI561725 than Hill81, Lewjain, Madsen, Eltan | Unidentified | *Unidentified* |
| P160 | REF8474 | 1.08E-02 | higher in PI561725 than Eltan, Madsen, Lewjain, PI561727, Hill81 | Unidentified | *Unidentified* |
| P160 | REF4342 | 4.80E-03 | lower in PI561727 & PI561725 than Lewjain, Madsen, Hill81; lower in PI561725 than Eltan | Unidentified | *Unidentified* |
| P160 | REF2943 | 9.99E-03 | lower in Madsen than Eltan, PI561725, PI561727 | Unidentified | *Unidentified* |

Supplementary Table 5 continued…

| **Dataset** | **OTUs** | **Adj. P-Value*** | **Comparison** | **Family** | **Scientific Name** |
| --- | --- | --- | --- | --- | --- |
| P160 | DENOVO66 | 9.65E-03 | higher in PI561727 & PI561725 than Eltan, Lewjain, Madsen; higher in PI561727 than Hill81 | Unidentified | *Unidentified* |
| P160 | DENOVO4008 | 8.97E-03 | higher in PI561725 than Hill81, PI561727, Eltan, Lewjain, Madsen | Unidentified | *Unidentified* |
| P160 | DENOVO21 | 1.27E-02 | lower in PI561727 than Madsen | Unidentified | *Unidentified* |
| P160 | REF8601 | 1.34E-02 | higher in Lewjain than PI561725 | Xanthomonadaceae | *Pseudoxanthomonas mexicana* |
| P35a | REF8481 | 3.58E-02 | higher in Madsen than PI561725 | Acidobacteriaceae | *Unidentified* |
| P35a | REF5301 | 3.42E-02 | higher in Madsen than PI561725 | Acidobacteriaceae | *Unidentified* |
| P35a | DENOVO442 | 5.98E-02 | higher in Madsen than PI561725 | Acidobacteriaceae | *Unidentified* |
| P35a | REF982 | 5.35E-02 | higher in Eltan than Hill81, PI561727 | Bradyrhizobiaceae | *Bradyrhizobium* sp. |
| P35a | REF2556 | 6.22E-02 | higher in Madsen than PI561725 | Burkholderiaceae | *Burkholderia* sp. |
| P35a | REF5075 | 3.81E-02 | lower in PI561725 than Eltan | Caulobacteraceae | *Mycoplana* sp. |
| P35a | REF994 | 4.30E-02 | higher in Eltan than Hill81, Lewjain, Madsen, PI561725 | Chitinophagaceae | *Chitinophaga* sp. |
| P35a | REF6907 | 1.96E-02 | higher in Eltan than Hill81, Lewjain, Madsen, PI561725 | Chitinophagaceae | *Chitinophaga arvensicola* |
| P35a | REF591 | 1.78E-02 | higher in Eltan than Hill81, Lewjain, Madsen | Chitinophagaceae | *Unidentified* |
| P35a | REF5077 | 3.73E-03 | higher in Eltan than Hill81 | Chitinophagaceae | *Chitinophaga* sp. |
| P35a | DENOVO799 | 4.35E-02 | higher in PI561725 than Eltan | Chitinophagaceae | *Flavisolibacter* sp. |
| P35a | DENOVO787 | 4.19E-02 | higher in Eltan than Hill81, Madsen, PI561725 | Chitinophagaceae | *Chitinophaga* sp. |
| P35a | DENOVO2591 | 4.04E-02 | higher in Eltan than Hill81, Lewjain, Madsen | Chitinophagaceae | *Chitinophaga arvensicola* |
| P35a | DENOVO1885 | 1.69E-02 | higher in Eltan than Hill81, PI561725 | Chitinophagaceae | *Chitinophaga* sp. |
| P35a | DENOVO1204 | 1.87E-02 | higher in Eltan than Hill81 | Chitinophagaceae | *Chitinophaga arvensicola* |
| P35a | REF254 | 6.41E-02 | higher in Eltan than Hill81, PI561725, PI561727 | Chthoniobacteraceae | *Unidentified* |
| P35a | REF1824 | 3.58E-02 | higher in Madsen than PI561725 | Comamonadaceae | *Variovorax* sp. |
| P35a | REF363 | 2.82E-02 | higher in Eltan than Hill81, Madsen; higher in PI561727 than Madsen | Hyphomicrobiaceae | *Devosia* sp. |

Supplementary Table 5 continued…

| **Dataset** | **OTUs** | **Adj. P-Value*** | **Comparison** | **Family** | **Scientific Name** |
| --- | --- | --- | --- | --- | --- |
| P35a | REF1968 | 3.43E-02 | higher in Hill81 than Eltan | Koribacteraceae | *Candidatus Koribacter* sp. |
| P35a | REF4277 | 1.83E-02 | higher in PI561727 than Lewjain | Moraxellaceae | *Unidentified* |
| P35a | REF317 | 3.62E-02 | higher in Eltan than Lewjain, Madsen, PI561727 | Mycobacteriaceae | *Mycobacterium* sp. |
| P35a | DENOVO299 | 1.34E-02 | higher in Eltan than Lewjain, Madsen | Mycobacteriaceae | *Mycobacterium* sp. |
| P35a | REF3941 | 1.66E-02 | higher in Eltan than Hill81, Madsen, PI561725;  higher in Lewjain than PI561727 | Nocardioidaceae | *Nocardioides* sp. |
| P35a | DENOVO2076 | 3.49E-02 | higher in PI561725 than Eltan | Nocardioidaceae | *Unidentified* |
| P35a | REF4070 | 4.54E-02 | higher in PI561725 than Eltan | Oxalobacteraceae | *Janthinobacterium* sp. |
| P35a | REF3578 | 5.80E-02 | higher in PI561725, Hill81 than Eltan | Oxalobacteraceae | *Janthinobacterium* sp. |
| P35a | REF2162 | 5.00E-02 | higher in PI561727 than Lewjain | Oxalobacteraceae | *Unidentified* |
| P35a | REF1729 | 1.57E-02 | higher in Madsen than PI561725 | Oxalobacteraceae | *Unidentified* |
| P35a | REF8227 | 3.77E-02 | higher in PI561725 than Madsen | Rhizobiaceae | *Rhizobium* sp. |
| P35a | REF7236 | 3.71E-02 | higher in PI561727 than Lewjain | Rhizobiaceae | *Unidentified* |
| P35a | REF4529 | 3.71E-02 | higher in PI561727 than Lewjain | Rhizobiaceae | *Rhizobium* sp. |
| P35a | REF4514 | 8.68E-03 | higher in Eltan than PI561727 | Rhizobiaceae | *Rhizobium* sp. |
| P35a | REF6072 | 4.54E-02 | higher in PI561725 than Eltan | Sphingobacteriaceae | *Unidentified* |
| P35a | REF520 | 6.40E-02 | lower in PI561727 than Lewjain | Sphingobacteriaceae | *Sphingobacterium multivorum* |
| P35a | REF3842 | 4.40E-02 | lower in Hill81 than Eltan | Sphingobacteriaceae | *Unidentified* |
| P35a | REF3136 | 4.31E-02 | lower in Madsen than Eltan, Hill81, PI561725 | Sphingobacteriaceae | *Unidentified* |
| P35a | DENOVO457 | 4.48E-02 | higher in Eltan than Hill81, Madsen | Sphingobacteriaceae | *Unidentified* |
| P35a | DENOVO4 | 1.15E-02 | lower in Hill81 than Eltan | Sphingobacteriaceae | *Unidentified* |
| P35a | DENOVO18 | 2.55E-02 | lower in Hill81 than Eltan, PI561725, Lewjain; lower in Madsen than Eltan | Sphingobacteriaceae | *Pedobacter* sp. |
| P35a | REF6703 | 5.45E-02 | lower in PI561725 than Eltan | Sphingomonadaceae | *Sphingomonas* sp. |
| P35a | REF890 | 1.54E-02 | higher in PI561725 & PI561727 than Lewjain, Madsen | Streptomycetaceae | *Unidentified* |
| P35a | REF5601 | 2.05E-02 | higher in PI561725 than Eltan, Madsen | Unidentified | *Unidentified* |

Supplementary Table 5 continued…

| **Dataset** | **OTUs** | **Adj. P-Value*** | **Comparison** | **Family** | **Scientific Name** |
| --- | --- | --- | --- | --- | --- |
| P35a | REF115 | 3.99E-02 | higher in PI561725 than Eltan, Madsen | Unidentified | *Unidentified* |
| P35a | DENOVO98 | 6.33E-02 | lower in Hill81 than Eltan | Unidentified | *Unidentified* |
| P35a | REF4467 | 5.09E-02 | higher in Hill81 than Eltan, PI561725, Lewjain, Madsen, PI561727 | Weeksellaceae | *Chryseobacterium* sp. |
| P35a | REF8806 | 1.83E-02 | lower in PI561727 than Lewjain | Xanthomonadaceae | *Luteimonas* sp. |
| P35a | REF4425 | 6.21E-02 | lower in PI561727 than Eltan | Xanthomonadaceae | *Stenotrophomonas sp.* |
| P35a | REF1794 | 5.77E-02 | lower in Lewjain than Eltan, PI561727 | Xanthomonadaceae | *Luteibacter rhizovicinus* |
| P35a | DENOVO42 | 5.78E-02 | lower in Lewjain than Eltan, PI561727 | Xanthomonadaceae | *Luteibacter rhizovicinus* |
| P35a | DENOVO1486 | 5.60E-02 | Lewjain than Eltan, PI561727 | Xanthomonadaceae | *Unidentified* |
| P35b | REF8284 | 4.37E-02 | lower in Eltan than Hill81, Madsen | Bradyrhizobiaceae | *Balneimonas* sp. |
| P35b | REF5019 | 4.86E-02 | higher in PI561725 than Lewjain | Burkholderiaceae | *Burkholderia* sp. |
| P35b | REF2457 | 2.21E-02 | higher in PI561725 than Hill81 | Burkholderiaceae | *Burkholderia* sp. |
| P35b | DENOVO37 | 3.60E-02 | higher in PI561725 than Lewjain | Burkholderiaceae | *Burkholderia* sp. |
| P35b | REF5685 | 4.98E-02 | higher in Lewjain than Eltan | Chitinophagaceae | *Flavisolibacter* sp. |
| P35b | REF1576 | 3.60E-02 | higher in Lewjain than PI561725 | Chitinophagaceae | *Unidentified* |
| P35b | DENOVO312 | 4.98E-02 | lower in Lewjain than Eltan | Chthoniobacteraceae | *Unidentified* |
| P35b | REF5854 | 4.12E-02 | lower in Hill81 than Eltan | Cytophagaceae | *Dyadobacter* sp. |
| P35b | DENOVO256 | 5.71E-02 | lower in Hill81 than Eltan | Cytophagaceae | *Dyadobacter* sp. |
| P35b | DENOVO321 | 1.40E-02 | higher in Lewjain than Eltan, PI561725 | Ellin517 | *Unidentified* |
| P35b | REF5687 | 3.42E-02 | higher in Eltan, Lewjain than Hill81 | Flavobacteriaceae | *Flavobacterium* sp. |
| P35b | REF1157 | 5.89E-02 | higher in Eltan than Hill81 | Microbacteriaceae | *Microbacterium* sp. |
| P35b | DENOVO36 | 3.68E-02 | higher in Eltan than Hill81, Lewjain | Microbacteriaceae | *Microbacterium* sp. |
| P35b | REF6840 | 5.23E-02 | higher in Lewjain than Hill81 | Opitutaceae | *Opitutus* sp. |
| P35b | REF4338 | 6.01E-02 | higher in Lewjain than PI561725, PI561727 | Opitutaceae | *Opitutus* sp. |
| P35b | DENOVO35 | 1.87E-02 | higher in Lewjain than Hill81 | Opitutaceae | *Opitutus* sp. |
| P35b | REF6555 | 1.53E-02 | higher in PI561725 than Eltan; higher in PI561725, Lewjain than Hill81 | Oxalobacteraceae | *Janthinobacterium* sp*.* |

Supplementary Table 5 continued…

| **Dataset** | **OTUs** | **Adj. P-Value*** | **Comparison** | **Family** | **Scientific Name** |
| --- | --- | --- | --- | --- | --- |
| P35b | REF5409 | 2.32E-02 | higher in PI561725 than Eltan | Oxalobacteraceae | *Janthinobacterium* sp. |
| P35b | REF409 | 5.22E-02 | higher in PI561725, Madsen than Eltan | Oxalobacteraceae | *Unidentified* |
| P35b | REF377 | 2.49E-02 | higher in Lewjain than Eltan | Oxalobacteraceae | *Unidentified* |
| P35b | REF2162 | 3.05E-02 | higher in PI561725, Eltan than Hill81; higher in Eltan than Lewjain, Madsen | Oxalobacteraceae | *Unidentified* |
| P35b | REF2754 | 4.74E-02 | higher in PI561727 than Madsen | Paenibacillaceae | *Paenibacillus* sp. |
| P35b | REF8663 | 2.80E-02 | lower in PI561727 than Madsen | Promicromonosporaceae | *Promicromonospora* sp. |
| P35b | REF3938 | 2.42E-02 | higher in PI561727 than Madsen, Hill81 | Pseudomonadaceae | *Pseudomonas* sp. |
| P35b | REF7639 | 2.09E-02 | higher in Lewjain than Eltan, PI561727, Hill81, PI561725 | Rhizobiaceae | *Agrobacterium* sp. |
| P35b | DENOVO1260 | 2.38E-02 | lower in Hill81 than Eltan | Rhizobiaceae | *Unidentified* |
| P35b | REF7915 | 3.82E-02 | higher in Hill81 than Lewjain | Sphingobacteriaceae | *Unidentified* |
| P35b | REF7655 | 5.07E-02 | higher in Eltan than Madsen, PI561725 | Sphingobacteriaceae | *Pedobacter* sp. |
| P35b | REF7015 | 4.35E-02 | higher in PI561725 than Hill81 | Sphingobacteriaceae | *Unidentified* |
| P35b | REF4083 | 5.60E-02 | higher in PI561725 than Hill81 | Sphingobacteriaceae | *Unidentified* |
| P35b | REF359 | 3.99E-02 | higher in Lewjain than Eltan, PI561727 | Sphingobacteriaceae | *Pedobacter* sp. |
| P35b | REF1367 | 3.71E-02 | higher in Lewjain than Hill81, PI561725 | Sphingobacteriaceae | *Pedobacter* sp. |
| P35b | DENOVO558 | 3.82E-02 | higher in Lewjain than Hill81 | Sphingobacteriaceae | *Pedobacter* sp. |
| P35b | DENOVO131 | 5.02E-02 | lower in Lewjain than Eltan, Hill81 | Sphingobacteriaceae | *Unidentified* |
| P35b | REF8789 | 2.83E-02 | higher in PI561725 than Hill81, Eltan | Sphingomonadaceae | *Novosphingobium* sp. |
| P35b | REF6703 | 5.07E-02 | higher in PI561725 than Eltan | Sphingomonadaceae | *Sphingomonas* sp. |
| P35b | REF6605 | 2.32E-02 | higher in PI561725 than Eltan | Sphingomonadaceae | *Sphingomonas* sp. |
| P35b | REF7887 | 5.24E-02 | higher in Eltan than Hill81, Lewjain, Madsen | Unidentified | *Unidentified* |
| P35b | REF760 | 1.87E-02 | higher in PI561727 than Madsen; higher in Eltan than Hill81, Madsen | Unidentified | *Unidentified* |
| P35b | REF1850 | 3.52E-02 | higher in Hill81 than Eltan, PI561725, Lewjain | Unidentified | *Unidentified* |

Supplementary Table 5 continued…

| **Dataset** | **OTUs** | **Adj. P-Value*** | **Comparison** | **Family** | **Scientific Name** |
| --- | --- | --- | --- | --- | --- |
| P35b | DENOVO66 | 5.60E-02 | higher in Hill81 than PI561725 | Unidentified | *Unidentified* |
| P35b | DENOVO398 | 3.61E-02 | lower in Hill81 than Eltan | Unidentified | *Unidentified* |
| P35b | DENOVO24 | 6.25E-02 | higher in Eltan than Hill81, PI561725 | Unidentified | *Unidentified* |
| P35b | DENOVO207 | 1.49E-02 | higher in Eltan than Hill81, Lewjain, PI561725 | Unidentified | *Unidentified* |
| P35b | DENOVO194 | 2.67E-02 | higher in Eltan than Hill81, Lewjain, PI561725 | Unidentified | *Unidentified* |
| P35b | REF6068 | 5.97E-02 | lower in Lewjain than Hill81 | Verrucomicrobiaceae | *Unidentified* |
| P35b | REF4103 | 3.69E-02 | higher in Lewjain than Hill81 | Verrucomicrobiaceae | *Unidentified* |
| P35b | REF8806 | 2.80E-02 | lower in PI561727 than Madsen | Xanthomonadaceae | *Luteimonas* sp. |
| P35b | REF6400 | 1.56E-02 | higher in Eltan than Hill81, PI561725 | Xanthomonadaceae | *Luteimonas* sp. |
| P35b | DENOVO2000 | 5.45E-02 | higher in Eltan than Hill81, PI561725 | Xanthomonadaceae | *Unidentified* |
| *Benjamini–Hochberg adjusted p-value | | | |  |  |


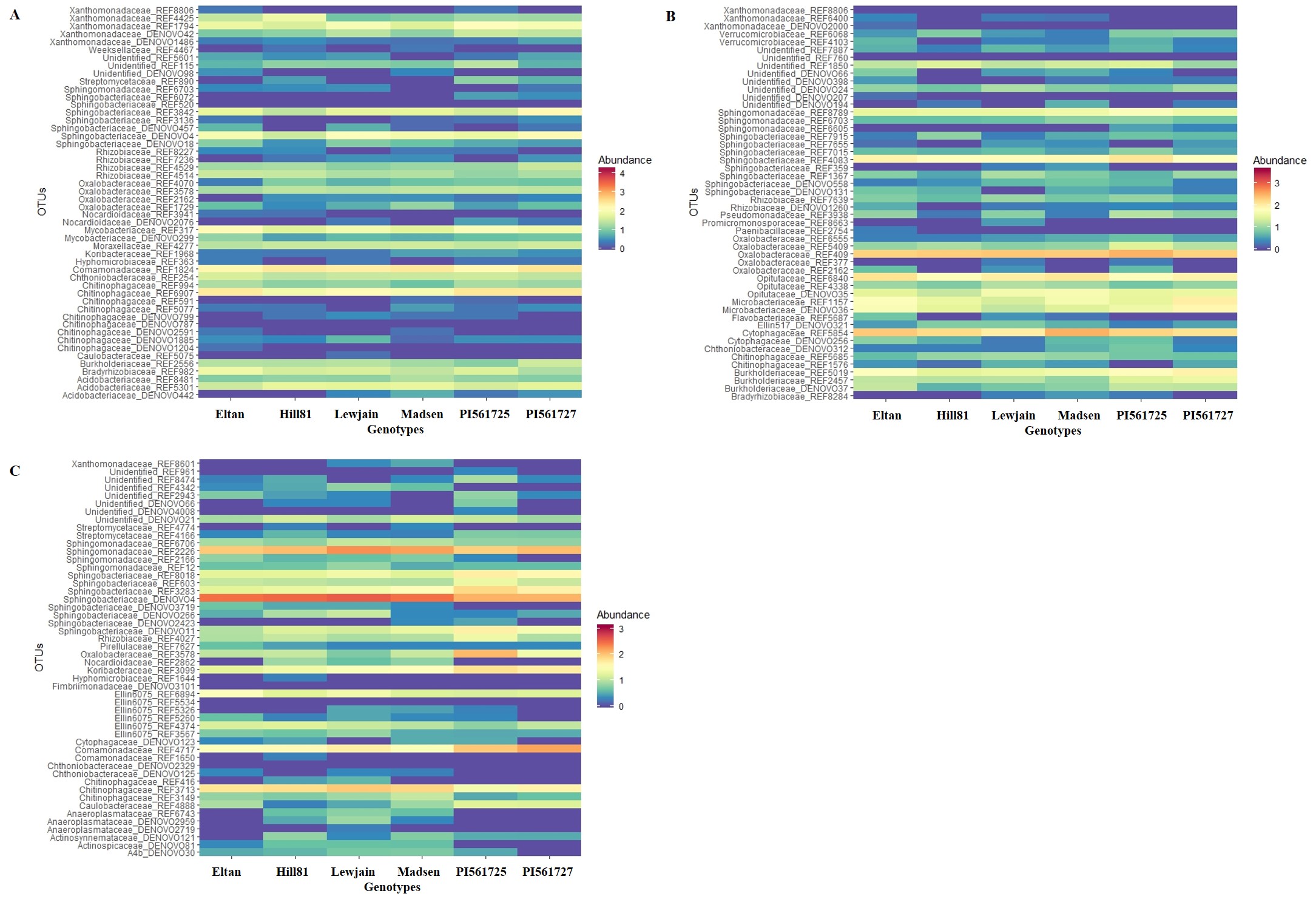
**Supplementary Figure 4:** **Differential abundance of top 50 bacterial OTUs across six winter wheat genotypes grown in Pullman soil under different growth chamber cycling lengths. (A)** 35- day cycles (Trial 1), **(B)** 35-day cycles (Trial 2), **(C)** 160-day cycles. Heatmap is based on normalized log(x+1) transformed counts and generated using DESeq2.


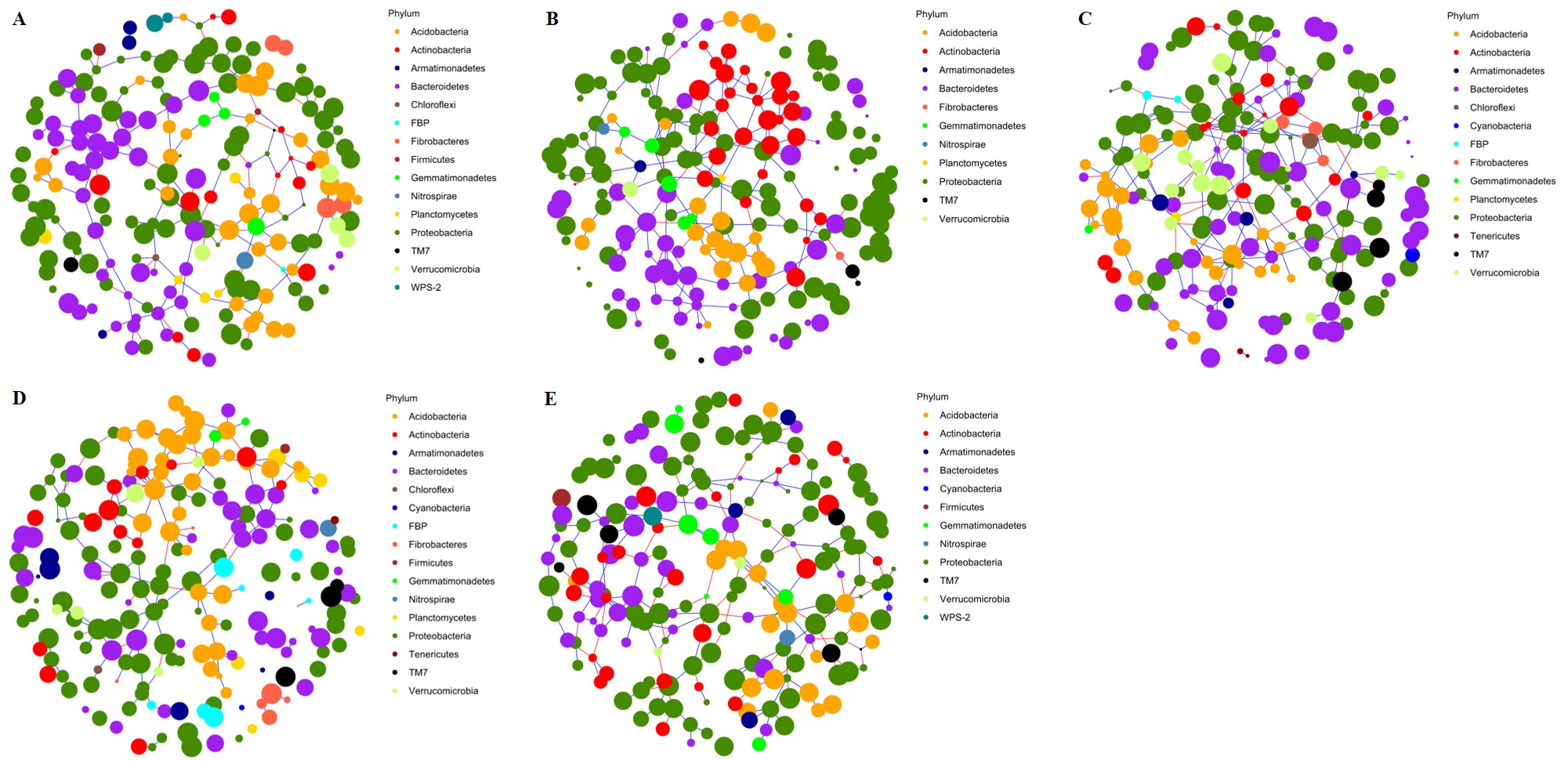


**Supplementary Figure 5:** **Network analysis of rhizosphere bacteriome by cycle length and from the field.** Sparse inverse covariance estimation network for **(A-D)** 28-, 35- (Trial 1), 35- (Trial 2), 160-day growth chamber cycle, **(E)** 2-year field cycling (Mahoney et al., 2017). Network generated after edge filtering using relative abundance of top 205 taxa as determined in SPIEC-EASI (SParse InversE Covariance Estimation for Ecological Association Inference).

**Supplementary Table 6:** Edge parameters of ecological networks estimated by SPEIC-EASI.

| **a. Edges before filtering** | |  |  |  |  |
| --- | --- | --- | --- | --- | --- |
| **Edges** | **P28** | **P35a** | **P35b** | **P160** | **2 Year Field** |
| Positive Edges | 312 | 440 | 438 | 336 | 283 |
| Negative Edges | 124 | 157 | 192 | 165 | 176 |
| Total Edges | 436 | 597 | 630 | 501 | 459 |
| % Positive | 71.56 | 73.70 | 69.52 | 67.07 | 61.66 |
|  |  |  |  |  |  |
| **b. Edges After Filtering** ^a^ | |  |  |  |  |
| **Edges** | **P28** | **P35a** | **P35b** | **P160** | **2 Year Field** |
| Positive Edges | 167 | 216 | 197 | 141 | 149 |
| Negative Edges | 52 | 23 | 49 | 32 | 80 |
| Total Edges | 219 | 239 | 246 | 173 | 229 |
| % Positive | 76.26 | 90.38 | 80.08 | 81.50 | 65.07 |
| ^a^ Edges with an absolute weight <0.1 were removed | | | | | |


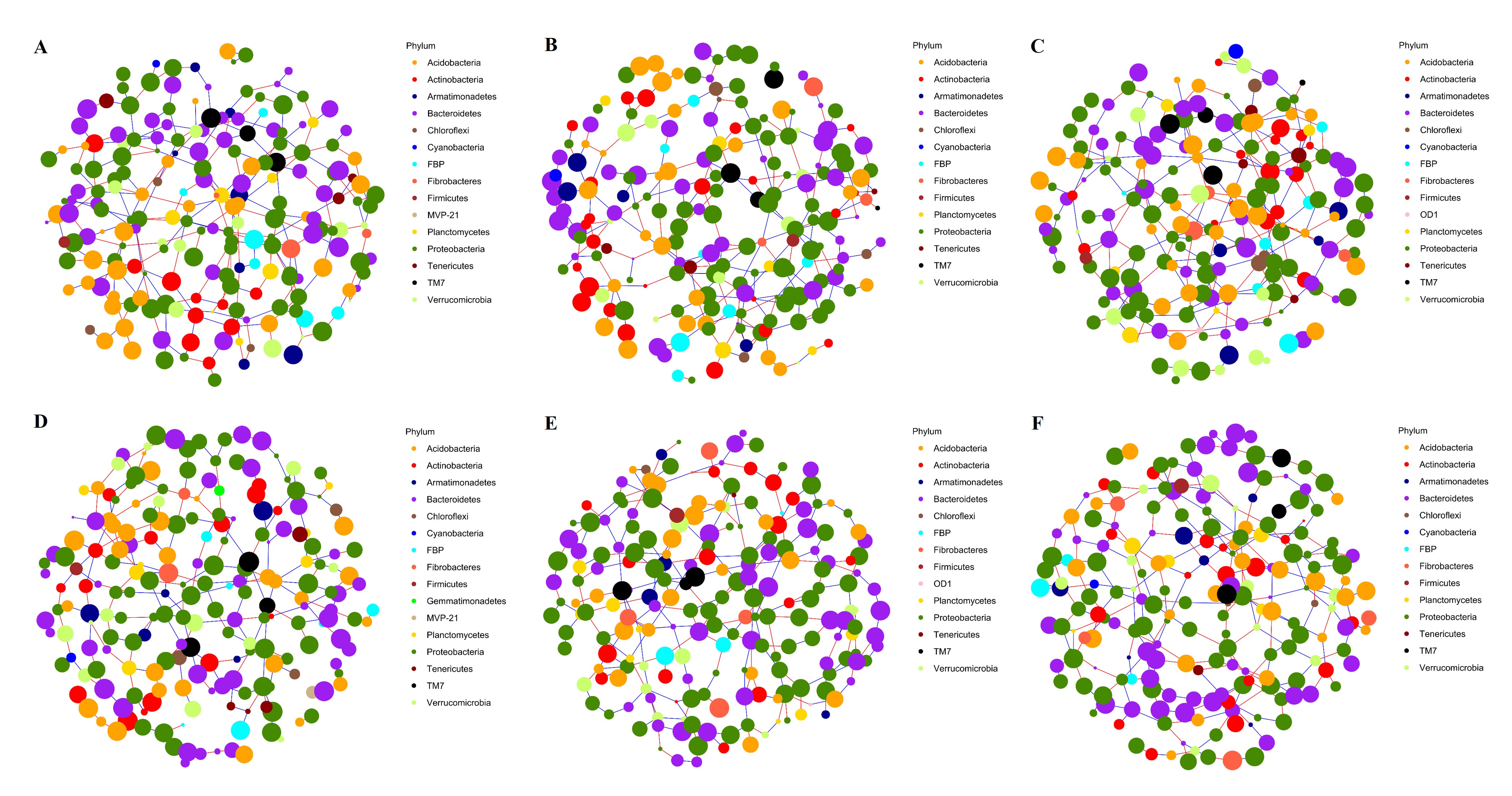


**Supplementary Figure 6:** **Network analysis of differentially abundant taxa in the rhizosphere bacteriome by genotype after 160 days of growth.** Sparse inverse covariance estimation network for **(A)** Eltan **(B)** Hill81 **(C)** Lewjain **(D)** Madsen **(E)** PI561725 **(F)** PI561727. Network generated after edge filtering using relative abundance of top 205 taxa as determined in SPIEC-EASI (SParse InversE Covariance Estimation for Ecological Association Inference).

**Supplementary Table 7:** Summary of edge parameters and roles of ecological networks estimated by SPEIC-EASI from the 160-day cycling experiment using differentially abundant OTUs.

| **a. Edges before filtering** |  |  |  |  |  |  |
| --- | --- | --- | --- | --- | --- | --- |
| **Edges** | **Eltan** | **Hill81** | **Lewjain** | **Madsen** | **PI561725** | **PI561727** |
| Positive Edges | 219 | 216 | 205 | 205 | 225 | 215 |
| Negative Edges | 192 | 191 | 188 | 172 | 178 | 195 |
| Total Edges | 411 | 407 | 393 | 377 | 403 | 410 |
| % Positive | 53.28 | 53.07 | 52.16 | 54.38 | 55.83 | 52.44 |
|  |  |  |  |  |  |  |
| **b. Edges After Filtering** ^a^ |  |  |  |  |  |  |
| **Edges** | **Eltan** | **Hill81** | **Lewjain** | **Madsen** | **PI561725** | **PI561727** |
| Positive Edges | 135 | 135 | 129 | 127 | 141 | 127 |
| Negative Edges | 109 | 105 | 119 | 104 | 106 | 119 |
| Total Edges | 244 | 240 | 248 | 231 | 247 | 246 |
| % Positive | 55.33 | 56.25 | 52.02 | 54.98 | 57.09 | 51.63 |
|  |  |  |  |  |  |  |
| **c. Modules and Network Roles** ^b^ | |  |  |  |  |  |
| **Module/ Roles** | **Eltan** | **Hill81** | **Lewjain** | **Madsen** | **PI561725** | **PI561727** |
| Module | 11 | 10 | 11 | 10 | 10 | 10 |
| Connector | 37 | 37 | 30 | 32 | 40 | 30 |
| Module Hub | 0 | 0 | 2 | 0 | 1 | 0 |
| Peripheral Hub | 1 | 2 | 3 | 2 | 2 | 1 |
| Network Hub | 0 | 0 | 0 | 0 | 0 | 0 |
| Peripheral | 0 | 0 | 0 | 0 | 114 | 132 |
| Ultra-Peripheral | 165 | 166 | 170 | 171 | 48 | 42 |
| Kinless | 2 | 0 | 0 | 0 | 0 | 0 |
| ^a^ Edges with an absolute weight <0.1 were removed  ^b^ Network modules determined using rnetcarto in R and roles described by Guimerà and Amaral (2005) and modification by Olesen et al., (2007) | | | | | | |

**
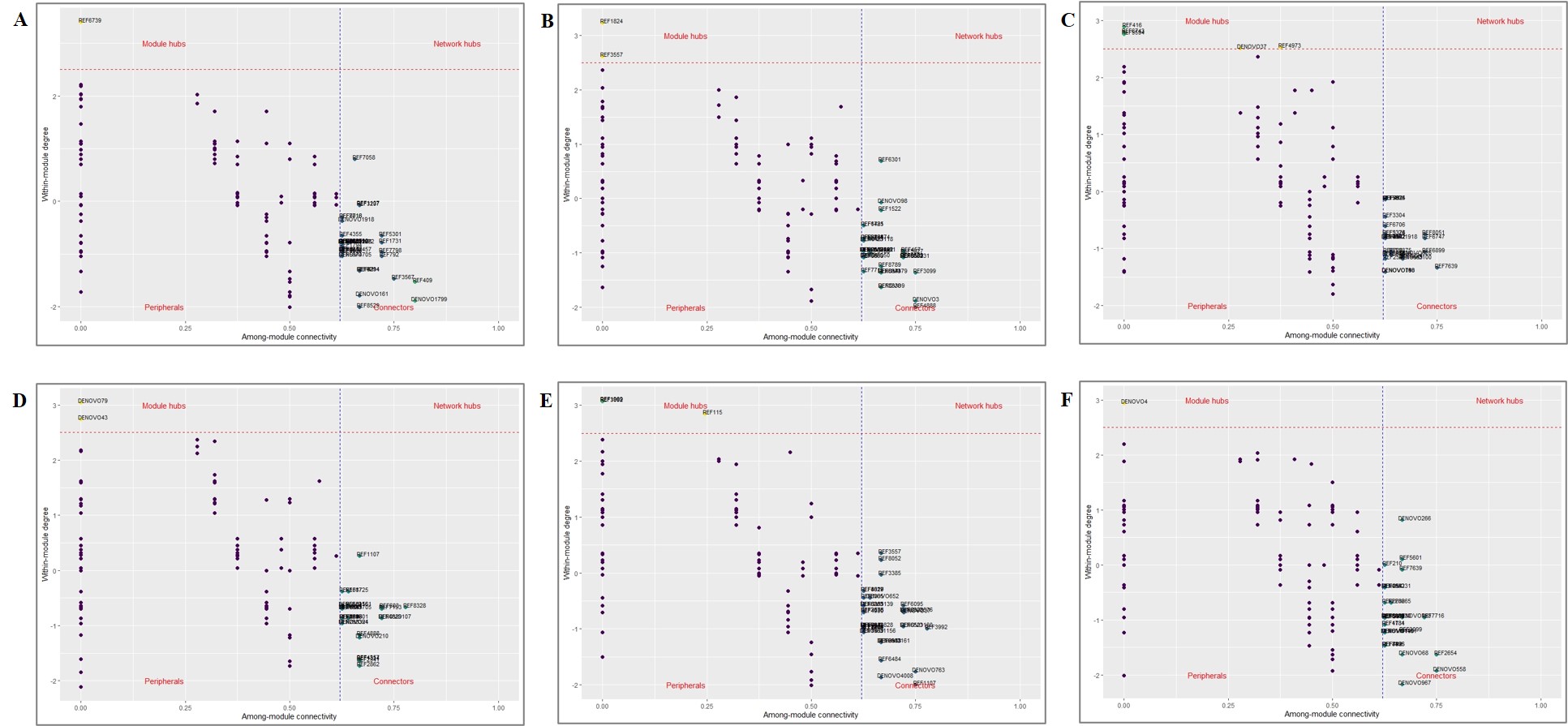
**

**Supplementary Figure 7:** **Network roles of differentially abundant taxa in the rhizosphere bacteriome of each genotype after 160 days of growth.** Bacterial taxa roles in covariance network of **(A)** Eltan **(B)** Hill81 **(C)** Lewjain **(D)** Madsen **(E)** PI561725 **(F)** PI561727. Network roles of top 205 taxa determined based on criteria described by Guimera and Amaral (2005) through rnetcarto package in R.

**Supplementary Table 8 :** Differentially abundant OTUs correlated with shoot length, shoot weight and root disease score in suppression assays.

| **A. Inoculated** | |  |  |  |  | |  | |  |  |  | |  | |  |  |  |
| --- | --- | --- | --- | --- | --- | --- | --- | --- | --- | --- | --- | --- | --- | --- | --- | --- | --- |
| **OTUs** | **Family** | **Shoot Length** | | | |  | | **Shoot Weight** | | | |  | | **Disease Score** | | | |
|  |  | **P-value ^a^** | **Corr. Coeff. ^b^** | **r^2^** |  | | **P-value ^a^** | | **Corr. Coeff. ^b^** | **r^2^** |  | | **P-value ^c^** | | **Corr. Coeff. ^d^** | **r^2^** |  |
| **REF3578** | Oxalobacteraceae | 0.014 | 0.386 | 0.127 |  | | 0.045 | | 0.315 | 0.076 |  | | 0.036 | | -0.328 | 0.130 |  |
| **REF4717** | Comamonadaceae | 0.006 | 0.429 | 0.163 |  | | 0.049 | | 0.309 | 0.073 |  | | 0.008 | | -0.406 | 0.140 |  |
| **REF1650** | Comamonadaceae | 0.002 | -0.485 | 0.215 |  | | 0.066 | | -0.289 | 0.060 |  | | 0.046 | | 0.313 | 0.063 |  |
| **REF2166** | Sphingomonadaceae | 0.256 | -0.184 | 0.008 |  | | 0.335 | | -0.154 | -0.001 |  | | 0.027 | | 0.344 | 0.091 |  |
| **REF8601** | Xanthomonadaceae | 0.462 | -0.120 | -0.012 |  | | 0.365 | | -0.145 | -0.004 |  | | 0.030 | | 0.340 | 0.009 |  |
| **REF4166** | Streptomycetaceae | 0.008 | 0.412 | 0.148 |  | | 0.018 | | 0.367 | 0.112 |  | | 0.097 | | -0.263 | 0.061 |  |
| **REF603** | Sphingobacteriaceae | 0.010 | 0.402 | 0.139 |  | | 0.160 | | 0.224 | 0.026 |  | | 0.332 | | -0.155 | 0.078 |  |
| **REF8018** | Sphingobacteriaceae | 0.018 | 0.371 | 0.115 |  | | 0.085 | | 0.272 | 0.050 |  | | 0.099 | | -0.261 | 0.053 |  |
| **REF3283** | Sphingobacteriaceae | 0.037 | 0.331 | 0.086 |  | | 0.107 | | 0.256 | 0.041 |  | | 0.287 | | -0.170 | 0.044 |  |
| **REF3099** | Koribacteraceae | 0.001 | 0.511 | 0.242 |  | | 0.002 | | 0.460 | 0.191 |  | | 0.067 | | -0.289 | 0.097 |  |
| **DENOVO2423** | Sphingobacteriaceae | 0.049 | 0.313 | 0.074 |  | | 0.113 | | 0.251 | 0.039 |  | | 0.291 | | -0.169 | 0.030 |  |
| **DENOVO11** | Sphingobacteriaceae | 0.007 | 0.420 | 0.155 |  | | 0.030 | | 0.339 | 0.092 |  | | 0.183 | | -0.212 | 0.057 |  |
| **REF6743** | Anaeroplasmataceae | 0.637 | -0.077 | -0.020 |  | | 0.881 | | 0.024 | -0.025 |  | | 0.699 | | 0.062 | -0.021 |  |
| **REF4027** | Rhizobiaceae | 0.102 | 0.262 | 0.044 |  | | 0.312 | | 0.162 | 0.001 |  | | 0.362 | | -0.146 | 0.038 |  |
| **REF961** | Unidentified | 0.313 | 0.164 | 0.001 |  | | 0.216 | | 0.198 | 0.014 |  | | 0.890 | | 0.022 | -0.008 |  |
| **DENOVO2719** | Anaeroplasmataceae | 0.215 | -0.200 | 0.015 |  | | 0.707 | | -0.061 | -0.022 |  | | 0.993 | | 0.001 | -0.024 |  |
| **DENOVO4** | Sphingobacteriaceae | 0.562 | -0.094 | -0.017 |  | | 0.683 | | -0.066 | -0.021 |  | | 0.202 | | 0.203 | 0.023 |  |
| **DENOVO2959** | Anaeroplasmataceae | 0.690 | -0.065 | -0.022 |  | | 0.988 | | 0.002 | -0.026 |  | | 0.712 | | 0.059 | -0.026 |  |
| **DENOVO81** | Actinospicaceae | 0.570 | -0.092 | -0.018 |  | | 0.739 | | -0.054 | -0.023 |  | | 0.199 | | 0.205 | 0.018 |  |
| **DENOVO828** | Weeksellaceae | 0.200 | 0.207 | 0.018 |  | | 0.717 | | 0.058 | -0.022 |  | | 0.671 | | -0.068 | 0.030 |  |

Supplementary Table 8 continued…

| **B. Uninoculated** | | |  |  |  | |  |  |  |  |
| --- | --- | --- | --- | --- | --- | --- | --- | --- | --- | --- |
|  | |  | **Shoot Length** | | | |  | **Shoot Weight** | | |
|  |  |  | **P-value ^a^** | **Corr. Coeff. ^b^** | **r^2^** | |  | **P-value ^a^** | **Corr. Coeff. ^b^** | **r^2^** |
| **REF3578** | | Oxalobacteraceae | 0.002 | 0.470 | 0.201 | |  | 0.000 | 0.585 | 0.325 |
| **REF4717** | | Comamonadaceae | 0.036 | 0.328 | 0.085 | |  | 0.021 | 0.360 | 0.108 |
| **REF1650** | | Comamonadaceae | 0.301 | -0.166 | 0.003 | |  | 0.188 | -0.210 | 0.019 |
| **REF2166** | | Sphingomonadaceae | 0.587 | -0.087 | -0.018 | |  | 0.057 | -0.300 | 0.067 |
| **REF8601** | | Xanthomonadaceae | 0.218 | -0.197 | 0.014 | |  | 0.638 | -0.076 | -0.020 |
| **REF4166** | | Streptomycetaceae | 0.165 | 0.221 | 0.024 | |  | 0.050 | 0.308 | 0.072 |
| **REF603** | | Sphingobacteriaceae | 0.005 | 0.435 | 0.168 | |  | 0.008 | 0.407 | 0.144 |
| **REF8018** | | Sphingobacteriaceae | 0.007 | 0.414 | 0.150 | |  | 0.002 | 0.468 | 0.199 |
| **REF3283** | | Sphingobacteriaceae | 0.014 | 0.381 | 0.123 | |  | 0.002 | 0.471 | 0.202 |
| **REF3099** | | Koribacteraceae | 0.294 | 0.168 | 0.003 | |  | 0.021 | 0.360 | 0.107 |
| **DENOVO2423** | | Sphingobacteriaceae | 0.118 | 0.248 | 0.037 | |  | 0.043 | 0.317 | 0.077 |
| **DENOVO11** | | Sphingobacteriaceae | 0.034 | 0.332 | 0.087 | |  | 0.021 | 0.361 | 0.108 |
| **REF6743** | | Anaeroplasmataceae | 0.263 | -0.179 | 0.007 | |  | 0.025 | -0.351 | 0.101 |
| **REF4027** | | Rhizobiaceae | 0.047 | 0.312 | 0.074 | |  | 0.047 | 0.312 | 0.074 |
| **REF961** | | Unidentified | 0.165 | 0.221 | 0.025 | |  | 0.049 | 0.309 | 0.072 |
| **DENOVO2719** | | Anaeroplasmataceae | 0.118 | -0.248 | 0.037 | |  | 0.022 | -0.358 | 0.106 |
| **DENOVO4** | | Sphingobacteriaceae | 0.299 | -0.166 | 0.003 | |  | 0.017 | -0.372 | 0.116 |
| **DENOVO2959** | | Anaeroplasmataceae | 0.178 | -0.215 | 0.022 | |  | 0.025 | -0.349 | 0.100 |
| **DENOVO81** | | Actinospicaceae | 0.068 | -0.288 | 0.059 | |  | 0.016 | -0.373 | 0.117 |
| **DENOVO828** | | Weeksellaceae | 0.021 | 0.359 | 0.106 | |  | 0.021 | 0.361 | 0.108 |
| ^a^ P-value based on Pearson method to test correlation | | | | |  |  |  |  |  |  |
| ^b^ Pearson correlation coefficient | | | | |  |  |  |  |  |  |
| ^c^ P-value based on Spearman method to test correlation | | | | |  |  |  |  |  |  |
| ^d^ Spearman correlation coefficient | | | | |  |  |  |  |  |  |

**Supplementary Table 9:** Metadata of raw sequence data in NCBI (BioProject PRJNA734707).

| **Experiment** | **SampleID** | **Wheat_Genotype** | **Accession Number** |
| --- | --- | --- | --- |
| 28-Day_GCCycle | FS15 | PI561725 | SAMN19518963 |
| 28-Day_GCCycle | FS17 | PI561727 | SAMN19518964 |
| 28-Day_GCCycle | FS19 | Lewjain | SAMN19518965 |
| 28-Day_GCCycle | FS27 | Lewjain | SAMN19518966 |
| 28-Day_GCCycle | FS28 | Hill81 | SAMN19518967 |
| 28-Day_GCCycle | FS35 | PI561727 | SAMN19518968 |
| 28-Day_GCCycle | FS37 | Eltan | SAMN19518969 |
| 28-Day_GCCycle | FS4 | Eltan | SAMN19518970 |
| 28-Day_GCCycle | FS45 | PI561725 | SAMN19518971 |
| 28-Day_GCCycle | FS46 | Madsen | SAMN19518972 |
| 28-Day_GCCycle | FS53 | PI561727 | SAMN19518973 |
| 28-Day_GCCycle | FS6 | Madsen | SAMN19518974 |
| 28-Day_GCCycle | FS60 | Madsen | SAMN19518975 |
| 28-Day_GCCycle | FS61 | PI561725 | SAMN19518976 |
| 28-Day_GCCycle | FS63 | Eltan | SAMN19518977 |
| 28-Day_GCCycle | FS64 | Lewjain | SAMN19518978 |
| 28-Day_GCCycle | FS72 | Hill81 | SAMN19518979 |
| 28-Day_GCCycle | FS73 | Lewjain | SAMN19518980 |
| 28-Day_GCCycle | FS84 | PI561727 | SAMN19518981 |
| 28-Day_GCCycle | FS86 | Hill81 | SAMN19518982 |
| 28-Day_GCCycle | FS87 | Madsen | SAMN19518983 |
| 28-Day_GCCycle | FS9 | Hill81 | SAMN19518984 |
| 35-Day_GCCycle | PSS01 | Madsen | SAMN19518985 |
| 35-Day_GCCycle | PSS02 | Eltan | SAMN19518986 |
| 35-Day_GCCycle | PSS03 | Lewjain | SAMN19518987 |
| 35-Day_GCCycle | PSS04 | PI561727 | SAMN19518988 |
| 35-Day_GCCycle | PSS06 | Hill81 | SAMN19518989 |
| 35-Day_GCCycle | PSS07 | Madsen | SAMN19518990 |
| 35-Day_GCCycle | PSS09 | PI561725 | SAMN19518991 |
| 35-Day_GCCycle | PSS10 | PI561727 | SAMN19518992 |
| 35-Day_GCCycle | PSS11 | Eltan | SAMN19518993 |
| 35-Day_GCCycle | PSS12 | Lewjain | SAMN19518994 |
| 35-Day_GCCycle | PSS13 | Hill81 | SAMN19518995 |
| 35-Day_GCCycle | PSS14 | PI561725 | SAMN19518996 |
| 35-Day_GCCycle | PSS15 | Hill81 | SAMN19518997 |
| 35-Day_GCCycle | PSS16 | Madsen | SAMN19518998 |
| 35-Day_GCCycle | PSS17 | Lewjain | SAMN19518999 |

Supplementary Table 9 continued…

| **Experiment** | **SampleID** | **Wheat_Genotype** | **Accession Number** |
| --- | --- | --- | --- |
| 35-Day_GCCycle | PSS18 | Madsen | SAMN19519000 |
| 35-Day_GCCycle | PSS19 | PI561725 | SAMN19519001 |
| 35-Day_GCCycle | PSS20 | Hill81 | SAMN19519002 |
| 35-Day_GCCycle | PSS21 | Eltan | SAMN19519003 |
| 35-Day_GCCycle | PSS23 | PI561727 | SAMN19519004 |
| 35-Day_GCCycle | PSS25 | Eltan | SAMN19519005 |
| 35-Day_GCCycle | PSS26 | PI561727 | SAMN19519006 |
| 35-Day_GCCycle | PSS27 | PI561725 | SAMN19519007 |
| 35-Day_GCCycle | PSS28 | Lewjain | SAMN19519008 |
| 35-Day_GCCycle | PSS30 | Lewjain | SAMN19519009 |
| 35-Day_GCCycle | PSS31 | Madsen | SAMN19519010 |
| 35-Day_GCCycle | PSS32 | Madsen | SAMN19519011 |
| 35-Day_GCCycle | PSS33 | PI561727 | SAMN19519012 |
| 35-Day_GCCycle | PSS35 | PI561725 | SAMN19519013 |
| 35-Day_GCCycle | PSS36 | Hill81 | SAMN19519014 |
| 35-Day_GCCycle | PSS37 | PI561725 | SAMN19519015 |
| 35-Day_GCCycle | PSS38 | PI561727 | SAMN19519016 |
| 35-Day_GCCycle | PSS39 | Lewjain | SAMN19519017 |
| 35-Day_GCCycle | PSS40 | Eltan | SAMN19519018 |
| 35-Day_GCCycle | PSS41 | Eltan | SAMN19519019 |
| 35-Day_GCCycle | PSS42 | Hill81 | SAMN19519020 |
| 35-Day_GCCycle | PSS43 | Madsen | SAMN19519021 |
| 35-Day_GCCycle | PSS44 | Hill81 | SAMN19519022 |
| 35-Day_GCCycle | PSS45 | Lewjain | SAMN19519023 |
| 35-Day_GCCycle | PSS47 | Hill81 | SAMN19519024 |
| 35-Day_GCCycle | PSS48 | PI561725 | SAMN19519025 |
| 35-Day_GCCycle | PSS49 | Eltan | SAMN19519026 |
| 35-Day_GCCycle | PSS50 | PI561727 | SAMN19519027 |
| 35-Day_GCCycle | PSS51 | Lewjain | SAMN19519028 |
| 35-Day_GCCycle | PSS52 | PI561727 | SAMN19519029 |
| 35-Day_GCCycle | PSS54 | PI561725 | SAMN19519030 |
| 35-Day_GCCycle | PSS55 | Eltan | SAMN19519031 |
| 35-Day_GCCycle | PSS56 | Madsen | SAMN19519032 |
| 35-Day_Trial2_GCCycle | PSR1 | Lewjain | SAMN19519033 |
| 35-Day_Trial2_GCCycle | PSR10 | PI561725 | SAMN19519034 |
| 35-Day_Trial2_GCCycle | PSR12 | Madsen | SAMN19519035 |
| 35-Day_Trial2_GCCycle | PSR13 | PI561727 | SAMN19519036 |

Supplementary Table 9 continued…

| **Experiment** | **SampleID** | **Wheat_Genotype** | **Accession Number** |  |
| --- | --- | --- | --- | --- |
| 35-Day_Trial2_GCCycle | PSR14 | Lewjain | SAMN19519037 |  |
| 35-Day_Trial2_GCCycle | PSR16 | Eltan | SAMN19519038 |  |
| 35-Day_Trial2_GCCycle | PSR17 | PI561725 | SAMN19519039 |  |
| 35-Day_Trial2_GCCycle | PSR18 | Lewjain | SAMN19519040 |  |
| 35-Day_Trial2_GCCycle | PSR19 | PI561725 | SAMN19519041 |  |
| 35-Day_Trial2_GCCycle | PSR2 | Eltan | SAMN19519042 |  |
| 35-Day_Trial2_GCCycle | PSR20 | Hill81 | SAMN19519043 |  |
| 35-Day_Trial2_GCCycle | PSR21 | PI561727 | SAMN19519044 |  |
| 35-Day_Trial2_GCCycle | PSR23 | Eltan | SAMN19519045 |  |
| 35-Day_Trial2_GCCycle | PSR24 | Madsen | SAMN19519046 |  |
| 35-Day_Trial2_GCCycle | PSR25 | Hill81 | SAMN19519047 |  |
| 35-Day_Trial2_GCCycle | PSR26 | Lewjain | SAMN19519048 |  |
| 35-Day_Trial2_GCCycle | PSR27 | Madsen | SAMN19519049 |  |
| 35-Day_Trial2_GCCycle | PSR28 | PI561727 | SAMN19519050 |  |
| 35-Day_Trial2_GCCycle | PSR29 | Madsen | SAMN19519051 |  |
| 35-Day_Trial2_GCCycle | PSR3 | Hill81 | SAMN19519052 |  |
| 35-Day_Trial2_GCCycle | PSR30 | Lewjain | SAMN19519053 |  |
| 35-Day_Trial2_GCCycle | PSR31 | Eltan | SAMN19519054 |  |
| 35-Day_Trial2_GCCycle | PSR33 | PI561725 | SAMN19519055 |  |
| 35-Day_Trial2_GCCycle | PSR34 | PI561727 | SAMN19519056 |  |
| 35-Day_Trial2_GCCycle | PSR35 | Madsen | SAMN19519057 |  |
| 35-Day_Trial2_GCCycle | PSR36 | PI561725 | SAMN19519058 |  |
| 35-Day_Trial2_GCCycle | PSR37 | Hill81 | SAMN19519059 |  |
| 35-Day_Trial2_GCCycle | PSR38 | Lewjain | SAMN19519060 |  |
| 35-Day_Trial2_GCCycle | PSR4 | PI561727 | SAMN19519061 |  |
| 35-Day_Trial2_GCCycle | PSR40 | Eltan | SAMN19519062 |  |
| 35-Day_Trial2_GCCycle | PSR41 | PI561727 | SAMN19519063 |  |
| 35-Day_Trial2_GCCycle | PSR42 | Hill81 | SAMN19519064 |  |
| 35-Day_Trial2_GCCycle | PSR43 | PI561727 | SAMN19519065 |  |
| 35-Day_Trial2_GCCycle | PSR44 | Eltan | SAMN19519066 |  |
| 35-Day_Trial2_GCCycle | PSR45 | Lewjain | SAMN19519067 |  |
| 35-Day_Trial2_GCCycle | PSR46 | Madsen | SAMN19519068 |  |
| 35-Day_Trial2_GCCycle | PSR48 | Hill81 | SAMN19519069 |  |
| 35-Day_Trial2_GCCycle | PSR49 | Madsen | SAMN19519070 |  |
| 35-Day_Trial2_GCCycle | PSR5 | Hill81 | SAMN19519071 |  |
| 35-Day_Trial2_GCCycle | PSR50 | PI561725 | SAMN19519072 | |
| 35-Day_Trial2_GCCycle | PSR51 | PI561727 | SAMN19519073 |  |

Supplementary Table 9 continued…

| **Experiment** | **SampleID** | **Wheat_Genotype** | **Accession Number** |
| --- | --- | --- | --- |
| 35-Day_Trial2_GCCycle | PSR52 | Eltan | SAMN19519074 |
| 35-Day_Trial2_GCCycle | PSR53 | Lewjain | SAMN19519075 |
| 35-Day_Trial2_GCCycle | PSR54 | PI561725 | SAMN19519076 |
| 35-Day_Trial2_GCCycle | PSR56 | Hill81 | SAMN19519077 |
| 35-Day_Trial2_GCCycle | PSR6 | Madsen | SAMN19519078 |
| 35-Day_Trial2_GCCycle | PSR7 | Eltan | SAMN19519079 |
| 35-Day_Trial2_GCCycle | PSR9 | PI561725 | SAMN19519080 |
| 160-Day_GCCycle | PE10 | Madsen | SAMN19519081 |
| 160-Day_GCCycle | PE11 | Eltan | SAMN19519082 |
| 160-Day_GCCycle | PE13 | PI561727 | SAMN19519083 |
| 160-Day_GCCycle | PE14 | Hill81 | SAMN19519084 |
| 160-Day_GCCycle | PE16 | Madsen | SAMN19519085 |
| 160-Day_GCCycle | PE17 | Eltan | SAMN19519086 |
| 160-Day_GCCycle | PE18 | PI561727 | SAMN19519087 |
| 160-Day_GCCycle | PE19 | Lewjain | SAMN19519088 |
| 160-Day_GCCycle | PE2 | Lewjain | SAMN19519089 |
| 160-Day_GCCycle | PE20 | PI561725 | SAMN19519090 |
| 160-Day_GCCycle | PE21 | PI561725 | SAMN19519091 |
| 160-Day_GCCycle | PE22 | PI561727 | SAMN19519092 |
| 160-Day_GCCycle | PE24 | Eltan | SAMN19519093 |
| 160-Day_GCCycle | PE25 | Hill81 | SAMN19519094 |
| 160-Day_GCCycle | PE26 | Madsen | SAMN19519095 |
| 160-Day_GCCycle | PE27 | Lewjain | SAMN19519096 |
| 160-Day_GCCycle | PE28 | Hill81 | SAMN19519097 |
| 160-Day_GCCycle | PE29 | Madsen | SAMN19519098 |
| 160-Day_GCCycle | PE3 | Lewjain | SAMN19519099 |
| 160-Day_GCCycle | PE30 | Lewjain | SAMN19519100 |
| 160-Day_GCCycle | PE31 | Eltan | SAMN19519101 |
| 160-Day_GCCycle | PE33 | PI561727 | SAMN19519102 |
| 160-Day_GCCycle | PE34 | PI561725 | SAMN19519103 |
| 160-Day_GCCycle | PE35 | Madsen | SAMN19519104 |
| 160-Day_GCCycle | PE37 | Hill81 | SAMN19519105 |
| 160-Day_GCCycle | PE38 | Lewjain | SAMN19519106 |
| 160-Day_GCCycle | PE39 | PI561727 | SAMN19519107 |
| 160-Day_GCCycle | PE4 | Eltan | SAMN19519108 |
| 160-Day_GCCycle | PE41 | PI561725 | SAMN19519109 |
| 160-Day_GCCycle | PE42 | Hill81 | SAMN19519110 |

Supplementary Table 9 continued…

| **Experiment** | **SampleID** | **Wheat_Genotype** | **Accession Number** |
| --- | --- | --- | --- |
| 160-Day_GCCycle | PE43 | Lewjain | SAMN19519111 |
| 160-Day_GCCycle | PE44 | Eltan | SAMN19519112 |
| 160-Day_GCCycle | PE45 | PI561727 | SAMN19519113 |
| 160-Day_GCCycle | PE48 | PI561725 | SAMN19519114 |
| 160-Day_GCCycle | PE5 | Hill81 | SAMN19519115 |
| 160-Day_GCCycle | PE50 | Madsen | SAMN19519116 |
| 160-Day_GCCycle | PE54 | Hill81 | SAMN19519117 |
| 160-Day_GCCycle | PE56 | Eltan | SAMN19519118 |
| 160-Day_GCCycle | PE6 | PI561727 | SAMN19519119 |
| 160-Day_GCCycle | PE7 | PI561725 | SAMN19519120 |
| 160-Day_GCCycle | PE8 | PI561725 | SAMN19519121 |
| 160-Day_GCCycle | PE9 | Madsen | SAMN19519122 |
| 2yr_Field (Mahoney et al. 2017) | Geno16 | PI561725 | SAMN19519123 |
| 2yr_Field (Mahoney et al. 2017) | Geno17 | PI561727 | SAMN19519124 |
| 2yr_Field (Mahoney et al. 2017) | Geno19 | Eltan | SAMN19519125 |
| 2yr_Field (Mahoney et al. 2017) | Geno2 | PI561725 | SAMN19519126 |
| 2yr_Field (Mahoney et al. 2017) | Geno20 | Madsen | SAMN19519127 |
| 2yr_Field (Mahoney et al. 2017) | Geno22 | Lewjain | SAMN19519128 |
| 2yr_Field (Mahoney et al. 2017) | Geno23 | Hill81 | SAMN19519129 |
| 2yr_Field (Mahoney et al. 2017) | Geno3 | PI561727 | SAMN19519130 |
| 2yr_Field (Mahoney et al. 2017) | Geno30 | PI561725 | SAMN19519131 |
| 2yr_Field (Mahoney et al. 2017) | Geno31 | PI561727 | SAMN19519132 |
| 2yr_Field (Mahoney et al. 2017) | Geno33 | Eltan | SAMN19519133 |
| 2yr_Field (Mahoney et al. 2017) | Geno34 | Madsen | SAMN19519134 |
| 2yr_Field (Mahoney et al. 2017) | Geno36 | Lewjain | SAMN19519135 |
| 2yr_Field (Mahoney et al. 2017) | Geno37 | Hill81 | SAMN19519136 |
| 2yr_Field (Mahoney et al. 2017) | Geno5 | Eltan | SAMN19519137 |
| 2yr_Field (Mahoney et al. 2017) | Geno6 | Madsen | SAMN19519138 |
| 2yr_Field (Mahoney et al. 2017) | Geno8 | Lewjain | SAMN19519139 |
| 2yr_Field (Mahoney et al. 2017) | Geno9 | Hill81 | SAMN19519140 |
